# Identification of Nicotinic Acetylcholine Receptor for N‐Acetylcysteine to Rescue Nicotine‐induced Injury Using Beating Cilia in Primary Tissue Derived Airway Organoids

**DOI:** 10.1002/advs.202407054

**Published:** 2024-11-24

**Authors:** Yichao Zheng, Qinyong Tian, Haowei Yang, Yongde Cai, Jiaxin Zhang, Yifen Wu, Shuo Zhu, Zuocheng Qiu, Yimin Lin, Jiangquan Hong, Yi Zhang, David Dockrell, Shaohua Ma

**Affiliations:** ^1^ Institute of Biopharmaceutical and Health Engineering Tsinghua Shenzhen International Graduate School (SIGS) Tsinghua University Shenzhen 518055 China; ^2^ Precision Medicine and Healthcare Research Centre Tsinghua‐Berkeley Shenzhen Institute (TBSI) Tsinghua University Shenzhen 518055 China; ^3^ Department of Cardiothoracic Surgery Zhangzhou Affiliated Hospital of Fujian Medical University Zhangzhou 363000 China; ^4^ Institute of Biopharmaceutical and Health Engineering State Key Laboratory of Chemical Oncogenomics Shenzhen International Graduate School Tsinghua University Shenzhen 518055 China; ^5^ Department of Internal Medicine Zhangzhou Affiliated Hospital of Fujian Medical University Zhangzhou 363000 China; ^6^ Key Laboratory of Rubber‐Plastics Ministry of Education/Shandong Provincial Key Laboratory of Rubber and Plastics Qingdao University of Science and Technology Qingdao 266042 China; ^7^ Guangdong Provincial Key Laboratory of Speed Capability Research Jinan University Guangzhou 510632 China; ^8^ Department of Respiratory Medicine and MRC Centre for Inflammation Research University of Edinburgh Edinburgh EH16 4TJ UK

**Keywords:** airway organoid, beating clia, NAC, nicotinic receptor

## Abstract

Smoking is one of the major contributors to airway injuries. N‐acetylcysteine (NAC) has been proposed as a treatment or preventive measure for such injuries. However, the exact nature of the smoking‐induced injury and the protective mechanism of NAC are not yet fully understood. Here, patient tissue‐derived airway organoids for modeling smoking‐induced injury, therapeutic investigation, and mechanism studies are developed. Airway organoids consist mainly of ciliated cells, together with basal cells, goblet cells, and myofibroblast‐like cells. The organoids display apical‐out and basal‐in polarity and are enriched in beating cilia, which are sensitive to smoking challenge and NAC treatment. An algorithm is developed to measure ciliary beating activity by analyzing the altered beating pattern of cilia in response to nicotine challenge and NAC treatment. Nicotinic acetylcholine receptors (nAChRs) expressed by airway organoids are involved in the mechanisms of nicotine‐induced injury through the nicotine‐nAChR pathway. In contrast to the common understanding that NAC has an antioxidative effect that mitigates airway damage, it is elucidated that NAC binding to nicotine can abolish the binding capacity of nicotine to nAChRs and thus prevent nicotine‐induced injury. This study focuses on the advances and potential of humanized organoids in understanding biological processes, mechanisms, and identifying therapeutic targets.

## Graphical abstract

Human primary tissue‐derived airway organoids can recapitulate the ciliary beating of the airway. The expression of nicotinic receptors in cilia plays a role in maintaining the ciliary beating. Nicotine decreases ciliary beating function by binding to the nicotinic receptors. N‐acetylcysteine reduces the toxicity of nicotine to airway organoids by suppressing nicotine binding to nicotinic receptors.

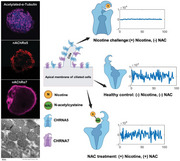

## 1 Introduction

Cigarette smoking is one of the major threats to global health. It is responsible for at least 8 million deaths annually, according to the World Health Organization (WHO). Tobacco and cigarette smoke contain a complex mixture of over 9500 chemicals,^[^ ^1^ ^]^ many of which can impair cell function as well as induce inflammation, gene mutation, and apoptosis.^[^ ^2^ ^]^ Nicotine, the primary compound in cigarettes, is harmful to the airway epithelium. It disrupts barrier function and weakens mucociliary clearance, therefore compromising the host defense against the invasion of respiratory pathogens.^[^ ^3^ ^]^ It also induces apoptosis and promotes tumorigenesis in a dose‐dependent manner.^[^ ^3^, ^4^ ^]^ The cytotoxicity of nicotine is largely dependent on the concentration and duration of nicotine exposure. It involves oxidative stress, DNA damage, activation of inflammation, programmed cell death, and disruption of cellular signaling.^[^ ^2^, ^4^, ^5^ ^]^

Nicotinic acetylcholine receptors (nAChRs) are widely distributed in the airway, especially in ciliated cells.^[^ ^6^ ^]^ In particular, nAChRα5 and nAChRα7 are highly expressed in human bronchial epithelial cells (HBECs) and smoking can alter their expression.^[^ ^6b^ ^]^ nAChRs exhibit a strong affinity for nicotine and have diverse physiological properties, including the regulation of mucociliary clearance by influencing ciliary beating and transepithelial ion transport processes.^[^ ^6^, ^7^ ^]^ Exposure to cigarette smoke can reduce ciliary beating frequency.^[^ ^8^ ^]^ However, the effects of nicotine on ciliary beating vary between different studies. In the study by K Maouche et al., nicotine did not significantly alter the ciliary beating frequency in airway epithelial cells.^[^ ^6a^ ^]^ However, Perniss and colleagues reported that acute nicotine exposure stimulated ciliary beating in the mouse trachea.^[^ ^9^ ^]^ The use of animal cells may bias the results because different species may have different sensitivities to nicotine treatment. In human lung and bronchial epithelial cells, nicotine challenge has been shown to decrease ciliary beating frequency.^[^ ^3^, ^10^ ^]^ In addition, the effects of nicotine on ciliary function may vary with dose and duration of exposure. In a human cohort, early and subtle exposure to cigarette smoke could provoke the respiratory defense mechanism by increasing the ciliary activity of airway epithelial cells.^[^ ^11^ ^]^ However, chronic nicotine exposure can significantly impair mucociliary clearance, which can be improved after smoking cessation.^[^ ^12^ ^]^ In our study, we investigated the effect of acute cigarette smoke or nicotine exposure on human airway epithelium.

N‐acetylcysteine (NAC) has both antioxidative and anti‐inflammatory properties.^[^ ^13^ ^]^ It has been widely used to treat respiratory diseases by reducing oxidative stress and inflammation.^[^ ^13a^ ^]^ It can attenuate smoking‐ or nicotine‐induced apoptosis by reducing the production of proinflammatory cytokines and free oxygen radicals.^[^ ^13b^ ^]^ In addition, NAC can improve mucociliary clearance by reducing mucus thickness due to its mucolytic properties.^[^ ^14^ ^]^ However, NAC was not found to directly affect ciliary beating frequency.^[^ ^15^ ^]^ It is unclear whether NAC can counteract nicotine‐induced ciliary dysfunction. To the best of our knowledge, there are no reports on the interaction between NAC and nAChRs.

Airway organoids are a powerful tool for studying respiratory diseases induced by smoking or nicotine. They can be developed from progenitor cells of primary airway tissue and consist of multiple airway‐specific cell types that self‐organize to form hollow structures.^[^ ^16^ ^]^ Airway organoids can recapitulate mucociliary clearance through ciliary beating and mucus secretion.^[^ ^16^, ^17^ ^]^ Airway organoids have been found to be sensitive to cigarette smoke challenges and have been used to identify novel treatments for smoking‐induced injury.^[^ ^18^ ^]^ However, to our knowledge, the effect of cigarette smoke or nicotine on the ciliary motility of the airway has never been studied beyond the air‐liquid interface (ALI) culture of bronchial epithelial cells.^[^ ^19^ ^]^ Airway organoids provide a more relevant model to study smoking‐ and nicotine‐induced ciliary dysfunction compared to submerged two‐dimensional (2D) cell culture and ALI culture. Therefore, we used primary airway tissue to establish airway organoids to model smoking‐ and nicotine‐induced injury. This airway organoid model recapitulates smoking‐ and nicotine‐induced ciliary dysfunction. The efficacy of NAC in ameliorating smoking‐ and nicotine‐induced injury will be evaluated. In addition, it was discovered that NAC can interact with nicotine through nAChRs and rescue nicotine‐induced ciliary dysfunction and injury.

## 2 Results

### 2.1 Construction of primary bronchial tissue‐derived motile airway organoids

**Figure 1:**
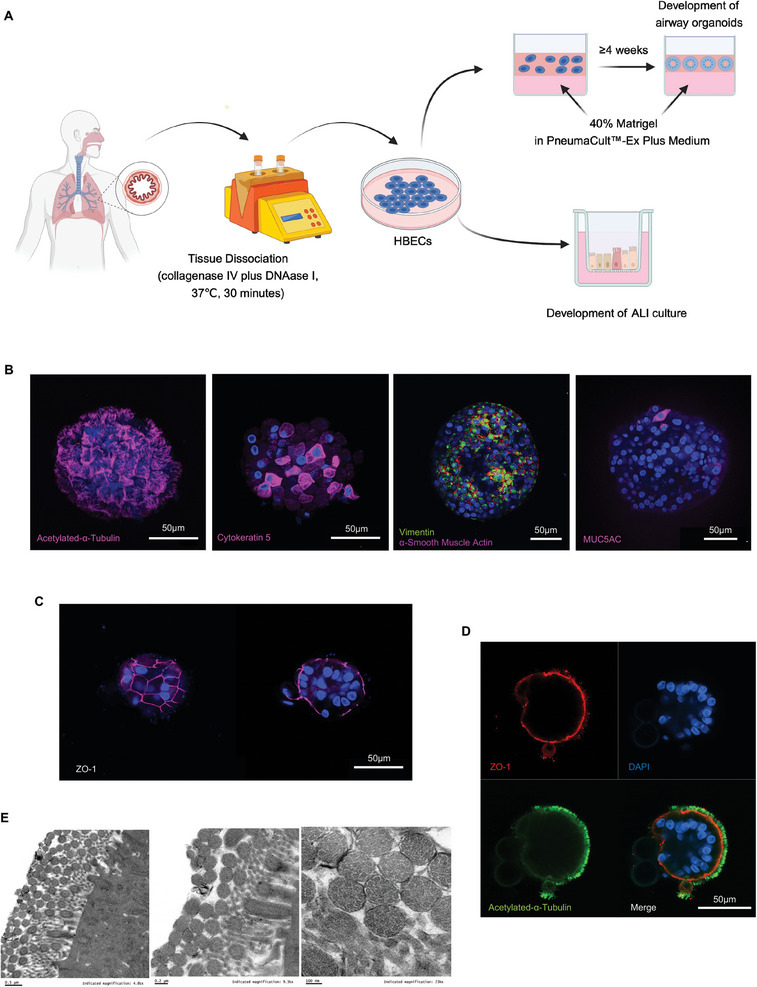
Establishment and characterization of airway organoids. A) Establishment of submerged 2D cell culture, ALI culture, and airway organoids. B) Airway organoids express a variety of cell markers, including acetylated‐α‐tubulin for ciliated cells, KRT5 for basal cells, MUC5AC for goblet cells, and αSMA/vimentin for myofibroblast‐like cells. C) Cells are tightly bound via the tight junction protein ZO‐1. D) Airway organoids display “apical‐out” polarization based on the relative position of Ac‐α‐Tub and ZO‐1. E) Motile cilia display a typical “9+2″ microtubule alignment using TEM. Abbreviations: TEM, transmission electron microscopy.

To develop a model for cigarette smoking research, we constructed airway organoids from human bronchial tissue‐derived epithelial cells (**Figure** **1**A). These cells can self‐organize into three‐dimensional (3D) bronchospheres after prolonged culture in vitro (Figure 1A). Long‐term culture of these bronchospheres promotes ciliary development, and therefore recapitulates the beating functionality of airways and generates the movement of organoids (Figure 1A; supplemental video S1). Airway organoids are composed of ciliated cells, basal cells, goblet cells, and myofibroblast‐like cells. These cell types are identified by the expression of specific markers: acetylated‐α‐tubulin (Ac‐α‐Tub) for ciliated cells, cytokeratin 5 (KRT5) for basal cells, mucin‐5AC (MUC5AC) for goblet cells, and α‐smooth muscle actin (αSMA)/vimentin for myofibroblast‐like cells (Figure 1B; Figures S1 and S2, Supporting Information). Ciliated cells are the predominant cell type in airway organoids and are responsible for promoting organoid movement in vitro through coordinated ciliary beating (Figure 1B; Figure S1, Supporting Information; supplemental video S1). Cells are tightly bound by the tight junction protein zonula occludens‐1 (ZO‐1) (Figure 1C). Airway organoids exhibit “apical‐out” polarization based on the relative position of Ac‐α‐Tub and ZO‐1 (Figure 1D; Figure S3, Supporting Information). Ac‐α‐Tub covers the exterior surface of the airway organoid, whereas ZO‐1 is located underneath the apical surface (Figure 1D; Figures S1 and S3, Supporting Information; supplemental video S2). The ultrastructure of cilia is characterized by a typical “9+2” microtubule alignment, consisting of 2 central microtubules surrounded by 9 outer microtubule doublets (Figure 1E; Figure S4, Supporting Information). These cilia beat synchronously and generate different types of motions, inducing lateral, rotary, and stationary motions (supplemental video S1). The ciliary beating activity of airway organoids was not significantly affected by the clinical characteristics of the donors, such as their smoking history and age (Figure S5, Supporting Information). Table S1 (Supporting Information) shows the clinical characteristics of the donors.

### 2.2 Cilia Motility as a Sensitive Biomechanical Indicator for Cigarette Smoke Toxicity

**Figure 2:**
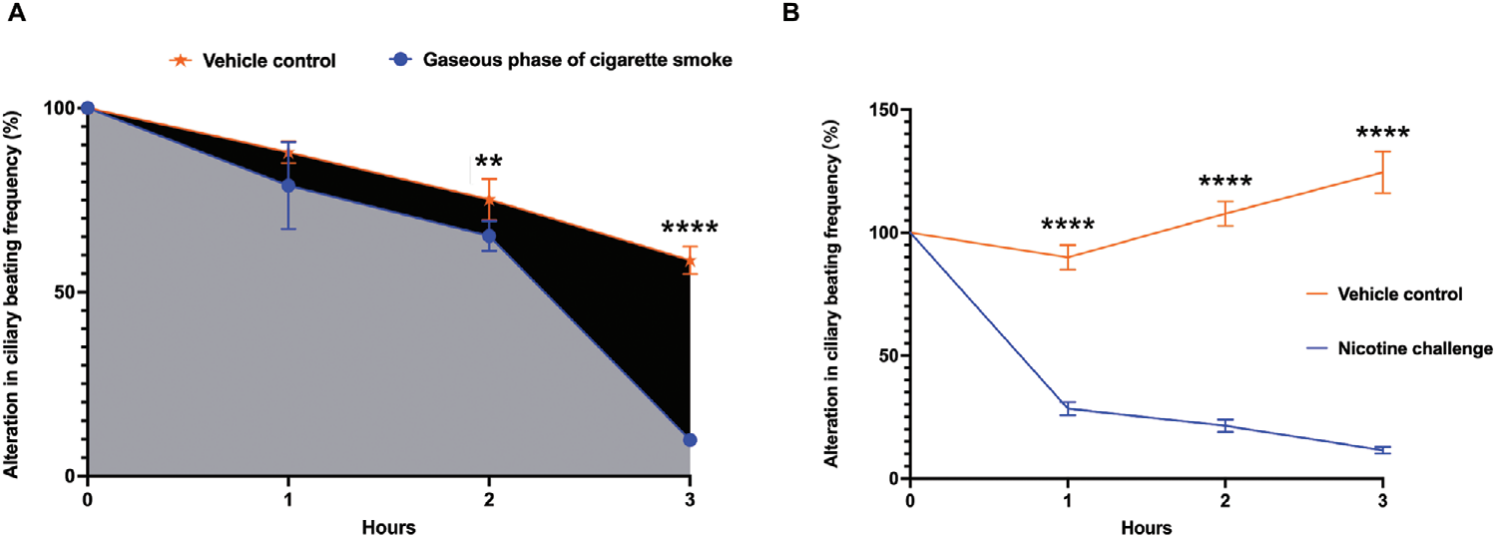
The sensitivity of beating cilia to the gaseous phase of cigarette smoke or nicotine challenge. A) Ciliary beating frequency decreases significantly in response to the 3‐hour of cigarette smoke challenge at 1 h intervals. The *P* values are 0.0987, 0.0052, and < 0.0001 at different time points (1, 2, and 3 h, respectively). B) Purified nicotine (5 mM) reduces ciliary beating. A *P* value of < 0.0001 is determined at various time points (1, 2, and 3 h, respectively). The line graph represents the mean and SD. The statistical significance between 2 different groups is determined using an unpaired *t‐*test, with a sample size of 6. Abbreviation: SD, standard deviation.

To assess the toxicity of cigarette smoke on motile cilia, we exposed airway organoids to the gaseous phase of cigarette smoke in an ALI setup. The results showed that the gaseous phase of cigarette smoke impaired ciliary functionality (**Figure** **2**A; supplemental video S3). Ciliary beating activity gradually decreased with prolonged exposure to the gaseous phase of smoke (Figure 2A). We further exposed airway organoids to cigarette condensate. Increasing the concentration of cigarette condensate and treatment time impaired the ciliary beating functionality of airway organoids (Figure S6, Supporting Information; supplemental video S4), further suggesting the sensitivity of cilia to cigarette smoke‐induced dysfunction. The proportion of live cells increased in airway organoids with higher ciliary beating activity, compared to organoids with lower ciliary beating activity (Figure S7, Supporting Information). The impairment of ciliary beating functionality occurs earlier than the appearance of cell death (Figure S7, Supporting Information; supplemental video S5), suggesting that ciliary beating activity may be a sensitive biomechanical marker for early detection of cigarette smoke‐induced injury.

We further investigated the sensitivity to cigarette condensate‐induced injury between the airway organoid model and traditional 2D cell culture. Exposure of the 2D culture of HBECs to different concentrations of cigarette condensate demonstrates the concentration‐ and time‐dependent toxicity of cigarette condensate to HBECs (Figure S8, Supporting Information). The percentage of live cells decreased while the percentage of apoptotic cells increased with increasing concentration of cigarette condensate and time of treatment (Figure S8, Supporting Information). Late apoptotic cells were the most common type of apoptotic cells and their proportions significantly increased in 2D culture of HBECs exposed to high concentrations of cigarette condensate for prolonged periods (Figure S8, Supporting Information). However, the same concentration of cigarette condensate did not significantly induce cell death in the airway organoids over the same time period (Figure S9, Supporting Information).

We further investigated the sensitivity of airway epithelial cells to the gaseous phase of cigarette smoke using the airway organoid model and 2D cell culture in the ALI setup. As shown in figure S10 (Supporting Information), the gaseous phase of cigarette smoke promptly induced apoptosis in the 2D culture of HBECs in the ALI setup. The proportion of early apoptotic cells significantly increased soon after exposure to the gaseous phase of cigarette smoke and entered the late apoptotic stage after prolonged exposure (Figure S10, Supporting Information). Compared to the 2D culture of HBECs, airway organoids were more resistant to this smoking‐induced apoptosis. Dead cells appeared later in the organoid model than in the 2D culture of HBECs (supplemental video S5).

Finally, we examined the alteration of ciliary beating activity upon exposure to purified nicotine, which was a major component of cigarette condensate determined by liquid chromatography‐mass spectrometry (LC‐MS) (Figure S11, Table S2, Supporting Information). As shown in Figure 2B, purified nicotine attenuated ciliary beating in a time‐dependent manner, suggesting a toxic effect of nicotine on ciliary beating function.

Overall, airway organoids exhibit physiologically representative features of the airway and are more resistant to cigarette smoke or nicotine‐induced cell death compared to 2D cell culture in submerged or ALI culture systems. This is probably because the compact 3D structure and mucociliary clearance functionality of airway organoids may prevent environmental hazards from penetrating the organoids. Beating cilia are susceptible to cigarette smoke or nicotine‐induced injury and thus may be a sensitive biomechanical indicator for early detection of environmental hazard‐induced injury. Loss of ciliary beating activity occurred earlier than the onset of cell death. Our study also suggests that airway organoids may be superior to 2D cell culture in recapitulating microstructural and biophysiological features of the airway under normal conditions or smoking exposure.

### 2.3 Evaluation of the protective Effect of NAC on Cilia Using Cigarette Smoke‐Exposed Airway Organoids

NAC is widely used in the clinical treatment of chronic obstructive pulmonary disease (COPD),^[^ ^13a^ ^]^ and has also been reported to protect against cigarette smoke‐induced epithelial damage. In both cases, the therapeutic or protective effect of NAC is thought to be attributed to its antioxidative properties. However, the clinical efficacy and underlying mechanism have not been fully elucidated by real‐time experimental observations in living humanized systems. This study investigated the therapeutic effects of NAC and its mechanism of action in airway organoids exposed to cigarette smoke and purified nicotine. To mimic smoking and NAC therapy in a real‐world scenario, we exposed airway organoids to the gaseous phase of cigarette smoke in the ALI setup after the treatment with aerosol NAC. We found that this inhaled NAC protected airway organoids from smoking‐induced ciliary dysfunction (Figure S12, Supporting Information; supplemental video S6). We further investigated the effects of liquid NAC on smoking‐induced injury. Pretreatment of airway organoids with liquid NAC prevented smoking‐induced ciliary dysfunction in a concentration‐dependent manner (Figure S13, Supporting Information; supplemental video S7). Increasing the concentration of NAC prolonged the duration of protection in the airway organoid model (Figure S13, Supporting Information; supplemental video S7). In addition, NAC treatment counteracted the toxicity of cigarette condensate and thus prevented cigarette condensate‐induced ciliary dysfunction (Figure S14; supplemental video S8).

We further investigated whether treatment with NAC could protect cells from cigarette condensate‐induced apoptosis. It was found that NAC treatment significantly increased the proportion of live cells and decreased the proportion of apoptotic cells in HBECs exposed to cigarette condensate within the 9‐hour time frame (Figure S15, Supporting Information). The protective effects of NAC were more pronounced when administered at high doses (≥ 10 mM) compared to low doses (≤ 1 mM) (Figure S15, Supporting Information). The proportion of live cells in HBECs was equivalent between the high‐dose NAC therapeutic group and the healthy group (Figure S15, Supporting Information). This indicated that NAC was effective in counteracting cigarette condensate toxicity. Importantly, increasing the concentration of NAC to 20 mM did not significantly increase its potential cytotoxicity to HBECs within the 9‐hour time frame of our study (Figure S15, Supporting Information). In addition, we did not observe apparent side effects of NAC on ciliary beating functionality in airway organoids (Figure S16; supplemental video S9). These findings suggest that airway organoids could be used to evaluate the efficacy and safety of drugs for the treatment of smoking‐induced injury.

### 2.4 NAC counteracts nicotine‐induced ciliary injury

**Figure 3:**
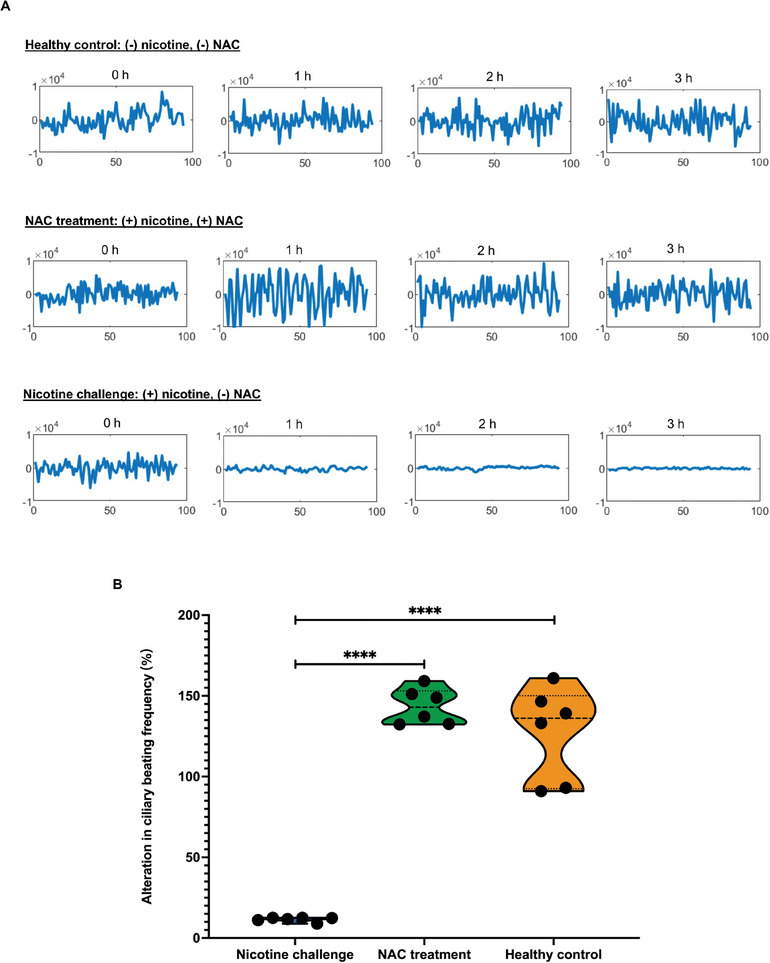
NAC prevents nicotine‐induced ciliary dysfunction. A) Nicotine diminishes the ciliary beating frequency as reflected by the attenuation of the fluctuation signals at different time points during the 3‐hour observation period, whereas NAC prevents this attenuation. B) Ciliary beating activity is significantly higher in the NAC therapeutic group than in the nicotine treatment group (*n* = 6) at the end of the experiment (3 h). Data are presented as mean and SD for each group (*n* = 6). Each symbol represents an individual. Ordinary ANOVA is used to determine the statistical difference between different groups with a *P* value of < 0.0001. Nicotine and NAC were both used at a final concentration of 5 mM. Abbreviation: ANOVA, analysis of variance.

Figure 2B shows that nicotine is a major toxicant that impairs ciliary beating functionality. Therefore, we investigated whether NAC is effective in mitigating or counteracting nicotine toxicity on airway organoids and their cilia. Our results indicate that NAC treatment can rescue nicotine‐induced ciliary dysfunction in airway organoids (**Figure** **3**; supplemental video S10). Ciliary beating frequency was higher in the NAC treatment group compared to the nicotine  group at different time points (Figure 3; supplemental video S10). This suggests the efficacy of NAC in preserving ciliary function after nicotine exposure. Taken together, these results suggest that NAC is effective in counteracting nicotine‐induced injury by improving ciliary function.

### 2.5 NAC interacts with nicotine and nicotinic receptors

**Figure 4:**
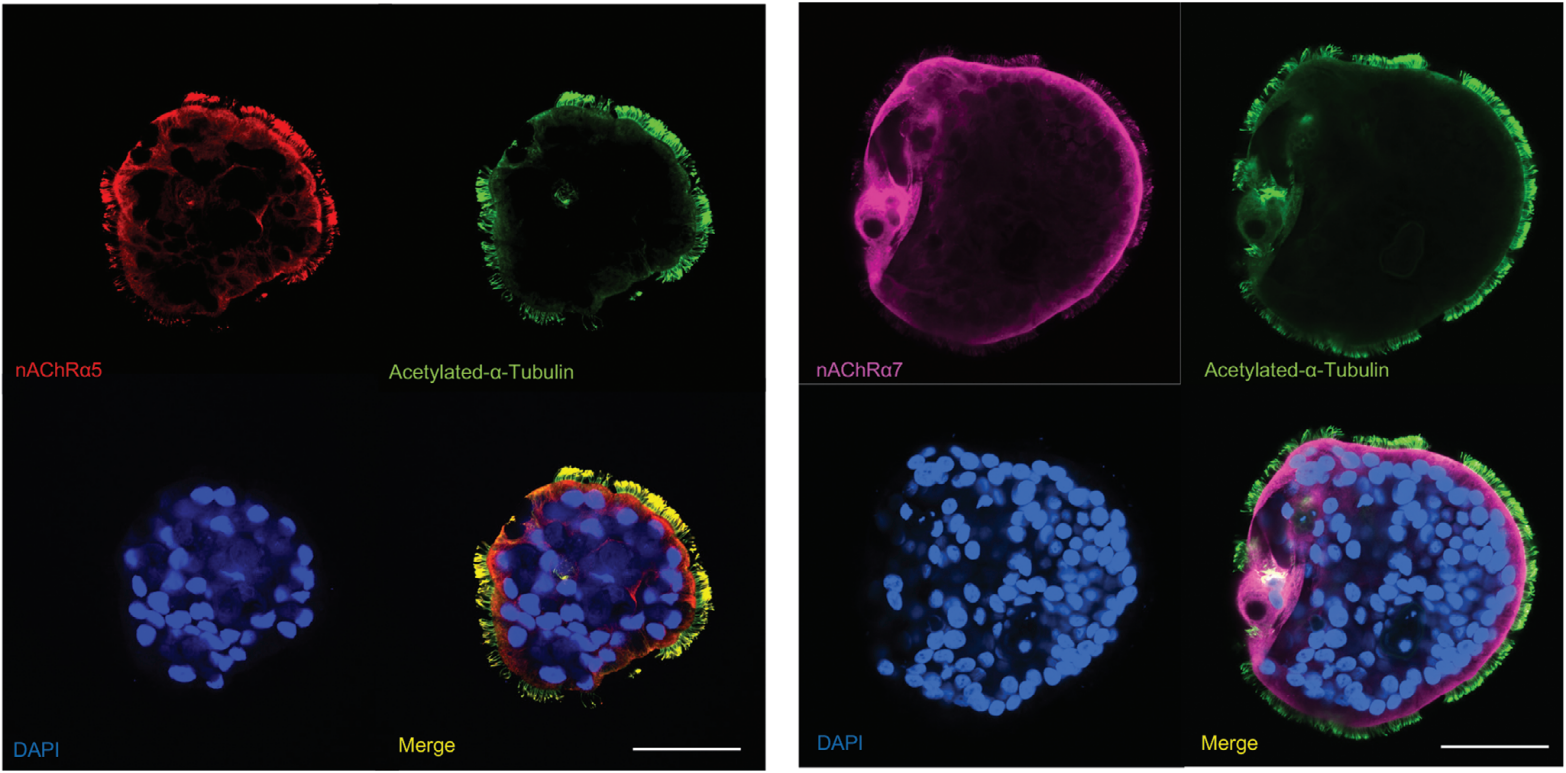
Airway organoids express nicotinic receptors. Cilia are visualized by acetylated‐α‐tubulin staining. nAChRα5 expression is enriched in the ciliary portion of the airway organoid, whereas nAChRα7 expression is more evident in the apical membrane of airway organoids than in the cilia. Scale bar, 50 µm.

**Figure 5:**
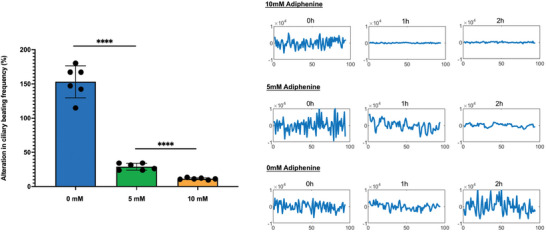
nAChR inhibitor suppresses the ciliary beating functionality. The nAChR inhibitor (adiphenine hydrochloride) reduces ciliary beating activity in a concentration‐dependent manner. The left figure shows the summary data of the alteration in ciliary beating frequency in airway organoids exposed to different concentrations of adiphenine hydrochloride at the end of the experiment (2 h). Data are presented with mean and SD for each group (*n* = 6). Each symbol represented a single individual. Ordinary ANOVA was used to determine the statistical difference between different groups, with a *p*‐value of < 0.0001. The right figure shows the representative data of the ciliary beating activity of organoids exposed to different concentrations of adiphenine hydrochloride at different time points.

**Figure 6:**
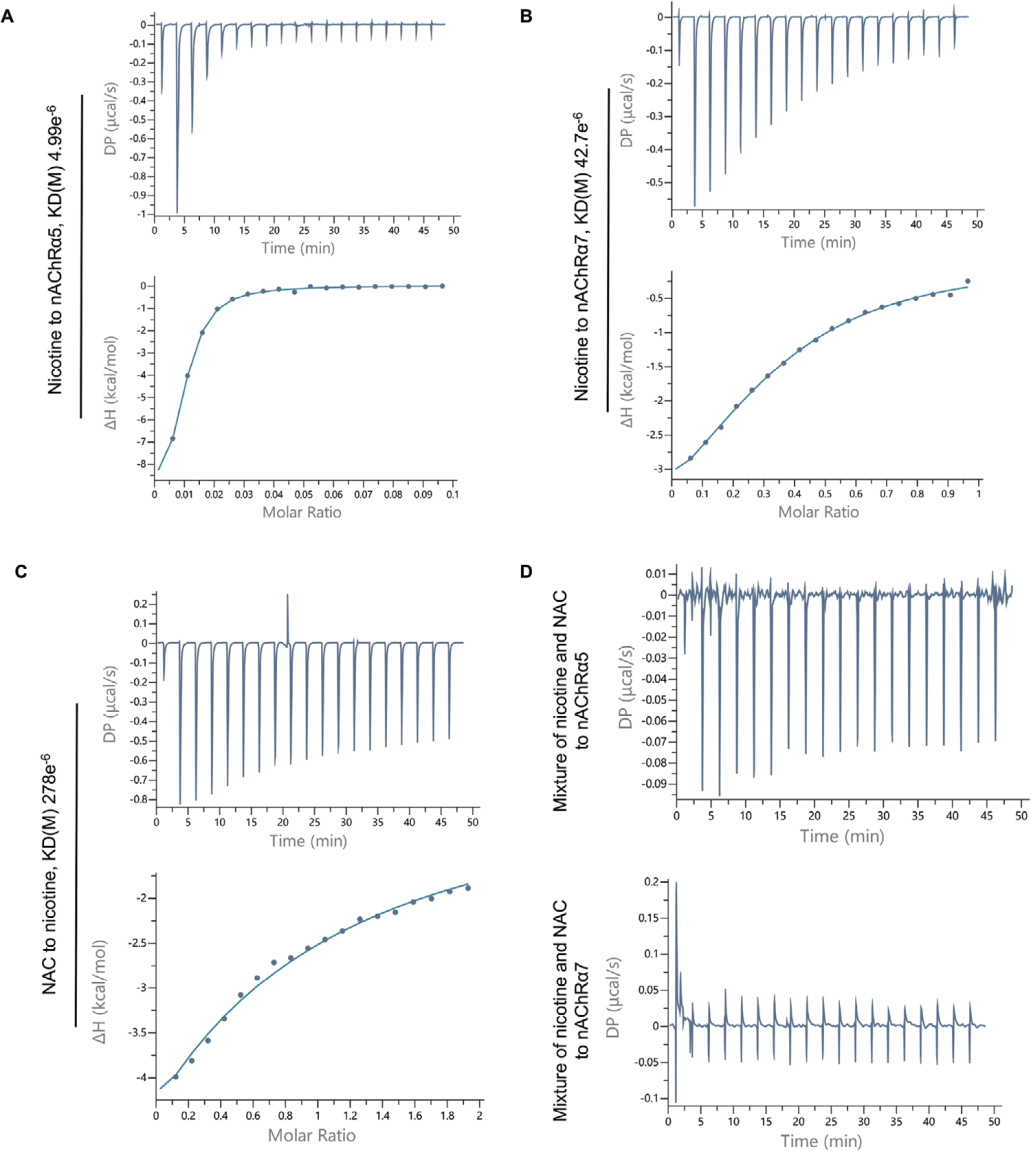
NAC interferes with the binding of nicotine to nicotinic receptors. A, B) The Binding of nicotine to nAChRα5 (A) and nAChRα7 (B) alter the thermodynamic parameters such as the enthalpy of reaction (ΔH) and differential power (DP) in a dose‐escalation manner, and therefore the affinity or equilibrium dissociation constant (KD) of 4.99e^−6^ and 42.7e^−6^ for nAChRα5 and nAChRα7 can be calculated respectively. C) NAC displays binding affinity to nicotine with an affinity or equilibrium dissociation constant (KD) of 278e^−6^. D) A mixture of NAC and nicotine loses the binding capacity to nAChRα5 and nAChRα7, because no apparent alterations in thermodynamic parameters were determined.

We further investigated the mechanisms of nicotine‐induced ciliary dysfunction and the role of NAC in preventing such injury. To elucidate the mechanisms of nicotine‐induced ciliary dysfunction, we first determined the expression and function of nicotinic receptors in airway organoids. nAChRα5 was predominantly enriched in the cilia of airway organoids, whereas nAChRα7 was more prominent in the apical membrane of ciliated cells than in the ciliary portion (**Figure** **4**; Figures S17–S19, Supporting Information). To investigate the functions of nAChRs in airway organoids, we used adiphenine hydrochloride to inhibit nAChRs. As expected, inhibition of nAChRs decreased ciliary beating in airway organoids (**Figure** **5**; supplemental video S11). This suggests that nAChRs play a role in ciliary beating. Nicotine could bind to different subtypes of nAChRs in cilia (**Figure** **6**A,B; Figure S20, Table S3, Supporting Information) and thus cause the dysfunction of nAChRs, resulting in a weakening of ciliary beating (Figure 2B). Taken together, these results collectively suggest that the nicotine‐nAChR pathway is highly involved in nicotine toxicity as reflected by ciliary beating functionality.

**Figure 7:**
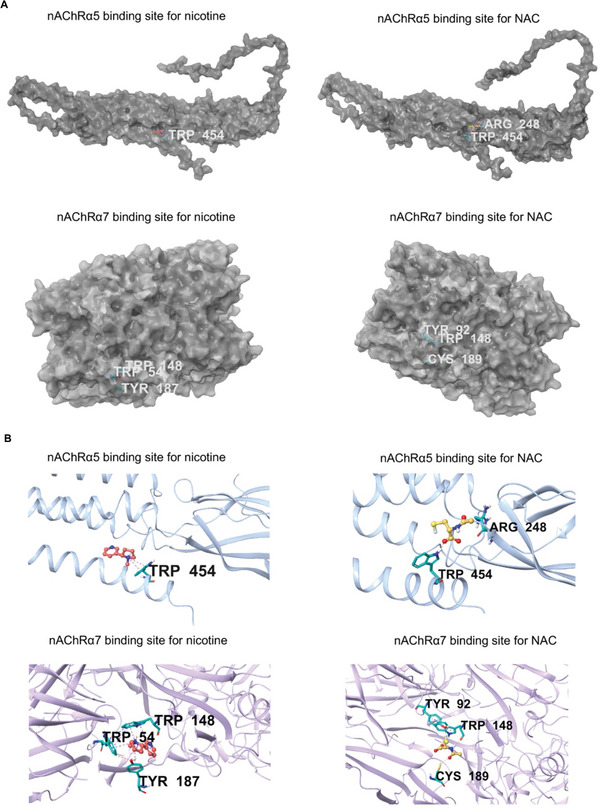
Computer docking simulations of molecular interactions. A) Molecular surface representation of the binding pocket of nAChRα5 and nAChRα7 in the crystal structure. Nicotine and NAC were represented by rod models. B) Cartoon representation of the binding sites of nAChRα5 and nAChRα7 for nicotine and NAC respectively.

**Figure 8:**
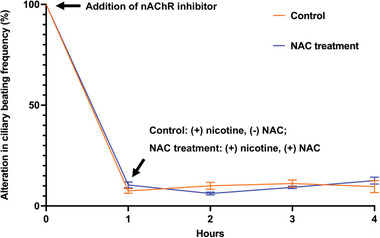
nAChRs are the potential therapeutic target for nicotine‐induced ciliary dysfunction. Adiphenine hydrochloride, an inhibitor of nAChRs, suppresses the ciliary beating activity in airway organoids. Inhibition of nAChRs abrogates the efficacy of NAC in rescuing nicotine‐induced ciliary dysfunction. Each group contains 6 individual data points (*n* = 6). Mann‐Whitney test was used for statistical analysis.

Next, we investigated the mechanism underlying the protective effect of NAC on nicotine‐induced ciliary dysfunction in airway organoids. NAC showed different binding capacities to different subtypes of nAChRs (Figure S21, Table S3, Supporting Information). However, the binding directions or angles are different for nicotine and NAC (**Figure** **7**). In addition, the other potential binding sites are different for nicotine and NAC according to the computer docking simulations (Figure 7). These results suggest that NAC does not directly compete with nicotine for nicotinic receptors. We then examined the direct interaction between NAC and nicotine. NAC could efficiently bind to nicotine, according to the change of thermodynamic parameters and absorption spectra during the molecular interaction (Figure 6C; Figure S22, Table S3, Supporting Information). Notably, the binding of NAC to nicotine could abolish the binding capacity of nicotine to nAChRs, and thus prevent nicotine‐induced ciliary dysfunction (Figure 6D; Figure S23, Supporting Information). In airway organoids where the function of nAChR is inhibited via Adiphenine, NAC treatment fails to prevent nicotine‐induced ciliary beating dysfunction (**Figure** **8**). These findings suggest that the protective effect of NAC is mediated through the action of nAChRs. Furthermore, NAC showed a binding capacity to cigarette condensate with a KD of 25.4e^−6^ (Figure S24, Table S3, Supporting Information). The molecular interactions between NAC and cigarette condensate were further confirmed by UV–Vis spectroscopy. The absorption spectra changed with increasing concentration of NAC in cigarette condensate (Figure S24, Supporting Information).

**Figure 9:**
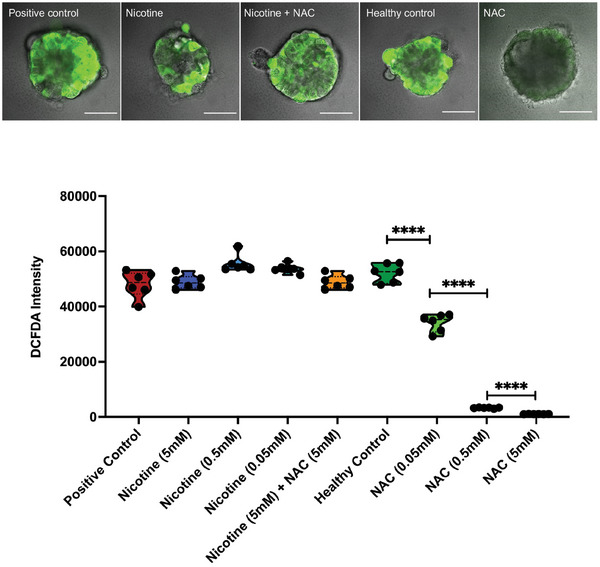
Effects of nicotine and NAC on ROS levels. DCFH‐DA was used to determine the levels of ROS in organoids under different treatments, such as Rosup (positive control), nicotine, NAC, and nicotine combined with NAC (top figure). Scale bar, 50 µm. Airway organoids from different groups were further dissociated into single organoid cells and ROS levels were assessed by determining the DCFDA intensity (bottom figure). Each group contains a total of 6 organoids from different donors. Statistical difference was determined by ordinary ANOVA. **** *p* < 0.0001. Abbreviations: DCFH‐DA, dichlorodihydrofluorescein diacetate.

Furthermore, we investigated the potential antioxidative property of NAC in mitigating nicotine‐induced ciliary dysfunction. As shown in **Figure** **9**, NAC could reduce reactive oxygen species (ROS) in healthy organoids. However, nicotine exposure did not further increase ROS levels in airway organoids and NAC did not significantly reduce ROS levels in nicotine‐exposed organoids. (Figure 9). These results suggest that inhibition of nicotine binding to nAChRs may be a major protective mechanism of NAC.

## 3 Discussion

In this study, we sought to elucidate the mechanisms underlying cigarette smoke‐induced airway injury. Our findings indicate that airway organoids provided a sensitive model for examining the effects of nicotine on airway epithelium. Our results demonstrate that ciliary dysfunction represents an early consequence of nicotine toxicity. The model was utilized to confirm the binding of nicotine to its receptor (nAChRs) in airway organoids. Moreover, we demonstrated that NAC could inhibit the interactions of nicotine with nAChRs and prevent ciliary dysfunction.

Cigarette smoke contains a variety of toxic substances, making it a direct respiratory hazard.^[^ ^1^, ^2^ ^]^ Nicotine, a primary component of cigarette smoke,^[^ ^1^ ^]^ can impair the mucociliary clearance of the airway. Prolonged exposure to nicotine has been demonstrated to cause dysfunction of the airway epithelium, which can mimic the absence or inactivation of nAChRs in mice or HBEC cultures. This suggests that nicotine may induce a malfunction of nAChRs.^[^ ^6a^ ^]^ However, previous studies have not identified a direct impact of nicotine on ciliary beating, nor have they provided direct evidence for the binding of nicotine to nAChRs.^[^ ^6a^ ^]^ In our study, we first reported that nicotine could bind to nAChRs, resulting in the dysfunction of nAChRs and ultimately leading to a weakening of ciliary beating. It is noteworthy that the exposure of airway organoids to nicotine did not result in a notable elevation in the level of ROS, as observed in our study. It may therefore be the case that the toxicity of nicotine to ciliary beating functionality is caused by a direct action of nicotine on nAChRs, rather than by the generation of ROS. However, additional research employing different concentrations of nicotine and exposure durations is necessary to ascertain the potential role of ROS in nicotine‐induced injury in other conditions.

nAChRs are ligand‐gated ion channels composed of 5 subunits that are permeable to calcium, potassium, and sodium ions.^[^ ^20^ ^]^ There are multiple subtypes of nAChRs with different subunit combinations. For example, nAChRα5 consists of both α and non‐α (β) subunits, whereas nAChRα7 consists entirely of α7 subunits.^[^ ^20^ ^]^ Opening of nAChR channels allows calcium and other ions to enter cells.^[^ ^20b^ ^]^ An increase in intracellular calcium levels facilitates the formation of the calcium‐calmodulin (Ca‐CAM) complex, which can activate adenylate cyclase (AC) and guanylate cyclase (GC).^[^ ^21^ ^]^ AC and GC can convert adenosine triphosphate (ATP) and guanosine triphosphate (GTP) into cyclic adenosine monophosphate (cAMP) and cyclic guanosine monophosphate (cGMP), respectively. The increased levels of cAMP and cGMP activate protein kinase A (PKA) and protein kinase G (PKG) in ciliary microtubules.^[^ ^21^, ^22^ ^]^ Airway cilia consist of 2 central singlet microtubules and 9 outer doublet microtubules that are coupled to dynein, forming a “9+2” axoneme.^[^ ^23^ ^]^ PKA and PKG can regulate axoneme sliding by modulating the phosphorylation of dynein, which in turn drives doublet microtubules to move along adjacent doublets, generating ciliary beating motility.^[^ ^21^, ^22^, ^23^ ^]^ In our study, inhibition of nAChRs suppressed the ciliary beating. This further suggests that nAChRs play an important role in the ciliary beating. Environmental factors affect ciliary motility by interacting with nAChRs. nAChRs show different responses to nicotine, depending on the subtype, concentration, and exposure time. For example, low concentration (100 µM) of nicotine can activate α3β4‐nAChRs and increase ciliary motility.^[^ ^9^ ^]^ However, prolonged nicotine exposure can cause nAChRα7 dysfunction.^[^ ^6a^ ^]^ In our study, we found that exposure of airway organoids to high concentrations of nicotine can abruptly impair ciliary beating activity.

NAC is a Food and Drug Administration (FDA)‐approved drug commonly prescribed for the treatment of smoking‐related bronchopulmonary diseases. The precise mechanism of action remains unclear. It is postulated to be attributable to the antioxidative and anti‐inflammatory properties of NAC.^[^ ^13^ ^]^ However, nicotine did not further increase ROS levels in airway organoids and NAC treatment did not significantly decrease ROS levels in nicotine‐treated organoids. These results suggest that the antioxidative effects of NAC may not be a primary mechanism of protection. Hence, we investigated the direct interaction between NAC and nicotine, which had not been previously documented. Our results showed that NAC can bind to nicotine and thereby counteract the toxicity of nicotine. Specifically, the binding of NAC to nicotine can prevent nicotine from binding to nAChRs, thereby reducing nicotine toxicity to cilia.

Airway organoids provide an excellent tool for understanding the mechanisms of smoking‐induced injury and identifying potential novel therapies.^[^ ^24^ ^]^ They are 3D cell cultures derived from primary human airway epithelial cells. The airway organoids established in this study consisted of ciliated cells, basal cells, goblet cells, and myofibroblast‐like cells, based on the expression of previously demonstrated markers,^[^ ^16^, ^17^, ^25^ ^]^ thus mimicking the cellular components, molecular phenotypes, ciliary structure, and function of the corresponding airway. Prolonged culture of airway organoids promoted the differentiation toward ciliated cells and cilia growth. To facilitate the investigation of the effects of environmental factors and drugs on ciliary functionality, an extracellular matrix (ECM)‐reduced approach was employed to reverse the polarization of the airway organoids. The hair‐like projection of the cilia indicated the “apical‐out” polarization of the airway organoids. This allows a direct exposure of cilia to environmental factors and drugs in conventional culture systems and facilitates the study of ciliary morphology and motility. Consistent with the ciliary structures of the airway,^[^ ^23^ ^]^ the cilia of airway organoids exhibit a “9+2” configuration of the axoneme, thereby establishing a structural foundation for ciliary beating. The ciliary beating motion indicated the functionality and maturation of the airway organoids established in this study. Importantly, we first identified the expression of nAChRs in the cilia of airway organoids. As previously shown, cilia can rely on nAChRs to receive environmental signals and thereby regulate their motility.^[^ ^26^ ^]^ Consistently, we found that both cigarette condensate and nicotine could bind to nAChRs and affect the ciliary motility of airway organoids. The airway organoids closely mimic the cellular components, molecular phenotypes, ciliary structures, and functionality of their counterparts. Therefore, they can be employed to examine the responses of the airway to environmental factors and to evaluate the effects of treatment.

Compared to 2D submerged and ALI cell cultures, airway organoids exhibit greater structural and physiological similarity to the airway. In particular, organoids derived from primary human tissue provide a genetically relevant model for studying human pathophysiology, reducing the need for animal experiments.^[^ ^27^ ^]^ Patient‐derived organoids can be used to evaluate the susceptibility of different individuals to cigarette smoke and the efficacy of treatment, thereby guiding precision medicine. The beating cilia of airway organoids provide an intuitive and sensitive biomechanical marker to assess the toxicity of environmental pollutants to the human airways and to test the efficacy of different therapeutics in preventing airway injury. In this respect, bright‐field and time‐lapse video microscopy allow label‐free and real‐time measurement of cilia functionality without compromising the vitality and function of airway organoids. Finally, we propose the use of ciliary beating frequency as a reliable marker to assess the motile function of airway organoids. However, the inherent differences between different airway organoids, such as size, shape, and cilia distribution, may result in different types of motility, as previously reported.^[^ ^17b^ ^]^ To minimize the impact of this inherent heterogeneity on the assessment of organoid functionality, we developed an algorithm to eliminate the effects of different movement types, thereby facilitating the quantification of ciliary beating activity. We validated our algorithm by using a conventional chemical staining. A decrease in ciliary beating activity determined by this algorithm is closely related to the subsequent increase in cell death determined by Calcein‐AM and propidium iodide (PI) staining. Therefore, a decrease in ciliary beating activity may be an early indicator of airway injury.

This study also suggests that ciliary dynamics may serve as a sensitive biomechanical marker of airway epithelial functionality. The change in ciliary beating frequency in airway epithelium exposed to environmental insults occurs prior to the onset of cell death. These findings have potential clinical applications. First, measuring ciliary beating activity in primary human airway organoids exposed to different environmental insults or drugs may help identify individuals who are susceptible to environmental factors or the side effects of drugs. Second, this approach may inform drug development and personalized medicine. Changes in ciliary beating activity may serve as biomarkers of individual response to specific treatments, thereby guiding precision medicine.

There are a few limitations in this study. First, although we have demonstrated that nicotine can bind to nAChRs and thereby induce ciliary dysfunction, the underlying mechanisms of nicotine‐induced nAChRs dysfunction remain to be elucidated. It is plausible that the binding of nicotine to nAChRs could impair the transepithelial ion transport processes in cilia, which might explain why the ciliary dysfunction occurs earlier than the onset of apoptosis. Second, the reason for the enhanced resilience of an intact airway organoid to environmental insults relative to single cells remains unclear. However, the mucociliary clearance function of airway organoids and the synergistic defense mechanism of different cell subsets may be involved. Thirdly, the binding affinity of other cigarette smoke compounds to nAChRs, their effects on the functionality of cilia, and the interactions between NAC and other cigarette smoke compounds have yet to be investigated. Given that NAC treatment can prevent airway organoids from cigarette smoke‐induced ciliary dysfunction and apoptosis, it is plausible that NAC may also counteract the toxicity of other cigarette smoke compounds. In addition, the primary objective of this study was to elucidate the interactive mechanism between nicotine and the airway epithelium, which is predominantly constituted of ciliated cells. However, it is possible that other cell subsets may also be implicated in nicotine‐induced injury, and further investigation may prove beneficial in the future. Finally, ciliary beating was employed as the primary marker of airway injury in this study. Previous studies showed that nicotine exposure impaired mucociliary clearance of airway epithelium, affected the extracellular remodeling, altered the cell components of the airway, activated a variety of cell signaling pathways, and influenced the expression of various cytokines and chemokines.^[^ ^6^, ^28^ ^]^ A comprehensive analysis of these alterations in airway organoids under nicotine exposure and NAC treatment will further our understanding of nicotine toxicity and the protective mechanism of NAC. These limitations indicate a need for further investigation to gain a comprehensive understanding of the adverse effects of smoking and air pollution, as well as to develop potential intervention strategies targeting the receptors for various environmental insults in the airway. The results of this study suggest that cigarette smoke‐induced early ciliary dysfunction may increase the risk of infection, stimulate inflammation, and consequently augment the toxicity to the airway epithelium. The administration of NAC in an aerosol formulation may prove an efficacious preventive measure against cigarette smoke‐induced epithelial injury.

## 4 Conclusion

The study demonstrated the value of organoids in elucidating disease mechanisms and identifying efficacious therapeutic or preventive interventions. Ciliary dysfunction serves as an early indicator of airway injury. The present study has identified potential new mechanisms underlying the protective effects of NAC against cigarette smoke or nicotine‐induced airway injury. In addition to the well‐recognized antioxidation and anti‐inflammatory properties of NAC, it was found that NAC could protect the airway from cigarette smoke‐induced injury by binding to cigarette smoke condensate or nicotine. Furthermore, NAC inhibited the binding of nicotine to nAChRs in the airway organoids. The cilia distribution and specific binding to both nicotine and NAC of nAChRα5, a previously unreported nicotinic receptor, suggest that it may play a more important role in these cases than the previously reported nAChRα7.^[^ ^6a^ ^]^

Primary tissue‐derived airway organoids are susceptible to cigarette smoke‐induced injury. This suggests that airway organoids are suitable for use in studies of environmental pollutant‐induced respiratory diseases and for therapeutic investigations. The beating cilia can be employed as a precise and sensitive biomechanical marker to assess the response of airway organoid cells to smoking and treatment. Ciliary dysfunction, indicated by a decrease in beating frequency, occurs prior to the onset of apoptosis. Therefore, the measurement of ciliary beating frequency can be employed to identify the toxicity of environmental factors to HBECs and to evaluate the efficacy of treatments at an early stage. The “apical‐out” polarization of airway organoids allows for the exposure of cilia to environmental toxicants in conventional culture devices, as well as the dynamic monitoring of motile alterations in cilia under video microscopy. Finally, we have identified nAChRα5, a subtype of nAChRs for NAC binding, that can rescue nicotine‐induced epithelial injury using beating cilia in primary tissue‐derived airway organoids. The findings of this study may facilitate the use of humanized organoids to explore new mechanisms and identify novel therapeutic targets.

## 5 Experimental Section

### Sample Collections and Ethics Statement

Study was conducted in accordance with the Declaration of Helsinki. This study was approved by the Ethics Committee of Zhangzhou Municipal Hospital of Fujian Province (Ref. 2023LWB342). Bronchial tissues were collected from a total of 10 patients who underwent surgery for lung disease with informed consent. The clinical characteristics of the participants are shown in Table S1 (Supporting Information). These surgically resected bronchial specimens were immersed in Roswell Park Memorial Institute 1640 (RMPI‐1640) medium supplemented with 10 µM Y27632 (STEMCELL Technologies), 100 µg mL^−1^ Primocin (InvivoGen), and 1% penicillin‐streptomycin‐amphotericin B solution (Solarbio, # P7630), and sent from the hospital to the laboratory within 24 h via cold chain shipping at 4–8 °C.

### Isolation and Culture of HBECs

Human bronchial tissues were dissected from the non‐diseased portions of surgically resected lung tissues. The bronchial tissues were extensively washed with cold phosphate‐buffered saline (PBS), and then the tissues were minced into small pieces before being transferred to tissue dissociation medium (RPMI‐1640 medium supplemented with 2 mg mL^−1^ collagenase IV and 0.1 mg mL^−1^ DNAase I). Tissue fragments were incubated in the tissue dissociation medium at 37 °C for 30 min, with intermittent agitation every 5 min. Tissue dissociation was terminated by the addition of RPMI‐1640 supplemented with 1% bovine serum albumin (BSA). The dissociated tissues were gently passed through a 100 µm strainer and the cells were collected for culture. HBECs were cultured in PneumaCult‐Ex Plus Medium (STEMCELL Technologies, #0 5040) at 37 °C under 5% CO2, and the medium was changed every 3 days until the cells reached ≈60% confluence when they were ready for passage. ACF Enzymatic Dissociation Solution (STEMCELL Technologies, #0 5426) was used to dissociate HBECs by incubating cells at 37 °C for 10 min, followed by gentle pipetting. ACF Enzyme Inhibition Solution (STEMCELL Technologies, #0 5426) was used to terminate this enzymatic dissociation. The cells were then pelleted to move the supernatant, resuspended in PneumaCult‐Ex Plus Medium at a concentration of 10^4 cells  mL^−1^, and incubated at 37 °C under 5% CO2. To prevent microbial overgrowth, Primocin, and penicillin‐streptomycin‐amphotericin B solution were added to the culture medium at a final concentration of 100 µg mL^−1^ and 1%, respectively. In addition, to prevent dissociation‐induced apoptosis, Y27632 was added to the culture medium at a final concentration of 10 µM during the first 3 days of culture for the primary culture and each passage.

### Establishment of ALI Culture

HBECs were harvested from submerged 2D cell culture to establish ALI culture by enzymatic dissociation as described above. HBECs were suspended in PneumaCult‐Ex Plus Medium at a concentration of 10^5^ cells mL^−1^ and plated onto 0.4 µm pore permeable membrane inserts loaded into a medium‐filled basal chamber of a transwell (Costar). The cells were cultured at 37 °C under 5% CO2 and the medium was changed every 2 days until the cells reached ≈80% confluence. The medium was then removed from the insert and the medium for the basal chamber of transwell was changed to PneumaCult‐ALI Medium (STEMCELL Technologies, #0 5001). The cells in ALI were cultured at 37 °C under 5% CO2 and the medium was changed every 2 days as described above. Cells were washed with PBS in the insert if excessive mucus was produced. Primocin and penicillin‐streptomycin‐amphotericin B solution were used to prevent microbial overgrowth as described above. Ciliated epithelium development was monitored by the bright‐field microscopy. The viability of the cells was determined through the use of Calcein‐AM and PI staining, or the FITC Annexin V Apoptosis Detection Kit with 7‐AAD (BioLegend, #640 922).

### Establishment and Maintenance of Primary Tissue‐Derived Airway Organoids

To establish airway organoids, HBECs were retrieved from 2D cell culture (passage 4) as described previously. Matrigel (Corning, #354 277) was diluted in cold PneumaCult‐Ex Plus Medium to prepare 40% and 5% cold Matrigel solution. The plate was coated with Matrigel by adding 40% Matrigel solution to the plate and incubated in a humidified incubator at 37 °C for 30 min to allow the Matrigel coating to solidify. The cells were suspended in 5% Matrigel solution and gently layered on the top of the Matrigel coating. The cells were incubated in a humidified incubator as described above, and the medium was changed every 3 days in the 5% Matrigel layer. Briefly, cells were gently removed from the 5% Matrigel layer without touching the Matrigel coating, pelleted to remove the supernatant, resuspended in fresh 5% Matrigel solution, and reseeded into the Matrigel‐coated plate. The cells self‐organize to form 3D bronchospheres within a one‐week period, and the beating cilia protrude from the exterior surface of the bronchospheres within a four‐week period, indicating the development of airway organoids. As previously described, both Primocin and penicillin‐streptomycin‐amphotericin B solution were used to inhibit microbial overgrowth in the culture medium.

### Preparation of Cigarette Condensate and Identification Of Compositions

Tobacco leaves were obtained from cigarette (CHINA TOBACCO FUJIAN INDUSTRIAL CO.LTD), and the cigarette condensate was prepared by soaking tobacco leaves in 10 times their own weight of RMPI‐1640 medium supplemented with 10% heat‐inactivated fetal bovine serum (FBS) overnight at 4 °C. Cigarette condensate was passed through a 40 µm strainer to remove tobacco leaves and other coarse particles. Small insoluble particles and bacteria were further eliminated by passing the cigarette condensate through a 0.22 µm microporous filter. The filtered cigarette condensate was stored at 4 °C and used for the subsequent experiment.

To identify the compositions in cigarette condensate, tobacco compounds were extracted in double distilled water (ddH2O) through maceration as described above. 500 µL of cigarette condensate was collected for subsequent analysis. The sample was spun at 13000 rpm for 10 min to pellet bacteria and residual insoluble particles, and the supernatant was collected to determine its composition. The compositions of cigarette condensate were determined by liquid chromatography‐mass spectrometry (LC‐MS) in accordance with the manufacturer's protocol. Analyses were performed in a Scientific Dionex UltiMate 3000 UHPLC instrument coupled to a high‐resolution Q‐Exactive mass spectrometer (Thermo Fisher Scientific, CA, USA). For liquid chromatography (LC), the column (C18, 2.1 mm × 100 mm, 1.9 µm particle size) were selected, and the mobile phase A and mobile phase B were prepared by adding formic acid to ddH2O and acetonitrile at a final concentration of 0.1%, respectively. The injection volume was 10 µL and the flow rate was 0.3 mL min^−1^. The detail of the LC gradient is shown in **Table** **1**. For mass spectrometry (MS), heated‐electrospray ionization (HESI) with the following parameters was utilized: probe heater 310 °C, capillary temperature 320 °C, sheath gas 30, auxiliary gas 10, IonSpray voltages 3 kV (positive ion mode) and 2.8 kV (negative ion mode). The data was acquired using the data‐dependent acquisition (DDA) mode. Stepped normalized collision energy (NCE) of 10, 28, and 35 eV were applied for higher‐energy collisional dissociation (HCD). The mass range was set to 100–1500 m z^−1^ with a resolution of 70000 (AGC 3E6, 200 ms maximum injection time) for MS1. Fragment analysis was performed in MS2 with a resolution of 17 500 (AGC 1E5, 50 ms maximum injection time).

**Table 1:** Liquid chromatography gradient.

Time [min]	Mobile phase A [%]	Mobile phase B [%]
0	90	10
10	0	100
15	0	100
17.1	90	10

The LC‐MS data were analyzed using Compound Discover (V3.2, Thermo Fisher Scientific, CA, USA) referenced to the public databases which included ChemSpider, ChEBI, ChEMBL, and mzCloud database. The chemical compounds were identified by matching the measured spectrum to the database of reference spectra and a total of 60 candidate compounds that displayed spectrally best matching against the reference spectra were selected. The peak area of the compound was proportional to its amount. The proportion of the peak area was calculated through dividing the peak area of interest by the total peak area. Therefore, the proportions of different compounds in the cigarette condensate were estimated.

### Nicotine/Cigarette Smoke Challenge and NAC Treatment

The cigarette was mounted on the top of a container where the smoke from the burning cigarette was collected by a suction pump. Therefore, ALI culture and airway organoids could be exposed to cigarette smoke in this device. Both ALI medium (PneumaCult‐ALI Medium) and airway organoid medium (PneumaCult‐Ex Plus Medium) were changed to RPMI‐1640 medium supplemented with 10% heat‐inactivated FBS prior to exposing the cells to cigarette smoke to minimize the effects of unknown medium compounds on the results. The cigarette was burned once an hour for a total of 3 h. Two different forms of NAC were used for the treatment of cigarette smoke‐induced injury. For NAC aerosol, an atomizer was used to generate NAC aerosol in the ALI culture prior to exposing the cells or organoids to the gaseous phase of cigarette smoke. This NAC aerosol treatment was applied once an hour for a total of 3 h. Fluimucil 100 mg mL^−1^ Solution for Inhalation was utilized to generate NAC aerosol. For NAC liquid, NAC was added to the culture medium prior to exposing the cells or organoids to the gaseous phase of cigarette smoke. The morphology and ciliary beating were recorded once an hour under the microscope (Nikon Ti2‐E).

For the submerged culture of HBECs or airway organoids, cigarette condensate or nicotine stock solution was first diluted in RPMI‐1640 medium supplemented with 10% heat‐inactivated FBS. The medium of the submerged HBEC culture and airway organoids was then changed to  cigarette condensate or nicotine at various concentrations before exposing the cells and organoids to the cigarette condensate or nicotine. Cells and organoids from different groups were examined at different time points to determine the ciliary beating, apoptotic rate, and morphological changes.

To determine the effect of NAC on the toxicity of cigarette condensate and nicotine, submerged HBEC cultures and airway organoids were exposed to the cigarette condensate and nicotine as described above in combination with NAC (Sigma‐Aldrich #A9165) at various concentrations. To assess the potential toxicity of NAC, submerged HBEC cultures and airway organoids were incubated in RPMI‐1640 medium supplemented with both NAC and 10% heat‐inactivated FBS. Bright‐field microscopy, immunofluorescence staining and flow cytometry were used to evaluate the ciliary functionality and cell viability at different time points under different conditions as described above.

In the selected experiment, airway organoids were incubated in normal saline supplemented with various concentrations of nAChR inhibitor (adiphenine hydrochloride, MCE HY‐B0379A) and the ciliary beating activity was examined under the microscope (Nikon Ti2‐E) at 1‐hour intervals until the cilia stopped beating. In a subgroup where cilia stopped beating following the adiphenine hydrochloride treatment, NAC was added to the culture of airway organoids in combination with nicotine at a final concentration of 5 mM, and ciliary beating was recorded under the microscope (Nikon Ti2‐E) at hourly intervals for another 3 h.

### Immunofluorescence Staining

Airway organoids were transferred to the adhesion microscope slide (CITOTEST #188 105) where immunofluorescence staining was performed under a microscope. Briefly, the organoids were first fixed with 4% paraformaldehyde for 10 min at room temperature (RT) and permeabilized with 0.1% Triton‐X‐100 for 15 min at RT. Nonspecific binding was blocked by incubating the organoids with 2% FBS in PBS for 1 h at RT. Second, organoids were incubated with primary antibodies at 1:50 dilution in 0.1% FBS overnight at 4 °C. Third, organoids were stained with fluorescence‐labeled secondary antibodies and 4′,6‐diamidino‐2‐phenylindole (DAPI) in the darkness for 1 h at RT at 1:300 and 1:5000 dilutions, respectively. The airway organoids were washed 3 times with PBS between each step as described above. Finally, a fluorescent mounting medium (Dako, #11 486 965) was added to the slide before the coverslip was applied. The primary antibodies used in this study included Ac‐α‐Tub (Sigma‐Aldrich, #T6793‐100UL), MUC5AC (Novus Biologicals, #NBP2‐15196), ZO‐1 (Invitrogen, #33‐9100), αSMA (ABclonal, # A17910), KRT5 (ABclonal, #A11396), vimentin (Affinity Biosciences, #BF8006), anti‐nAChRα5 (abcam, ab259859) and anti‐nAChRα7 (abcam, ab216485) antibodies. The secondary antibodies included Alexa Fluor 488 donkey anti‐mouse secondary antibody (Abcam, #ab150105), Alexa Fluor 568 donkey anti‐rabbit secondary antibody (Abcam, #ab175470), Alexa Fluor 647 donkey anti‐rabbit secondary antibody (Abcam, # ab150075), and Alexa Fluor 647 donkey anti‐mouse secondary antibody (Abcam, # ab150107).

For live and dead staining, airway organoids were stained with Calcein‐AM and PI (Yeasen Biotechnology, #40747ES80) for 30 min at 37 °C at 1:1000 and 3:1000 dilution in assay buffer respectively, in accordance with the manufacture's protocol. Then, organoids were washed 3 times with assay buffer to remove excess dye. Immunofluorescence images were captured using a Nikon AX confocal microscope with 60X and 100X oil immersion objectives.

In the selected experiment, highly intelligent and sensitive (HIS)‐structured illumination microscopy (SIM) (Fusion X, Guangzhou CSR Biotech Co. Ltd) was used to capture the structure of cilia, as well as the expression of nAChRα5 and nAChRα7. A 100X/1.49 NA oil immersion objective (Nikon) was chosen to capture the images, and the SIM images were collected and analyzed in accordance with a previously described protocol.^[^ ^29^ ^]^ The image quality was further improved using sparse deconvolution as described previously.^[^ ^30^ ^]^

### Flow Cytometry

HBECs were collected from ALI culture and submerged 2D cell culture by ACF enzymatic dissociation, as described previously. Live and apoptotic cells were identified in different groups at different time points using the FITC Annexin V Apoptosis Detection Kit with 7‐AAD (BioLegend, #640 922), in accordance with the manufacturer's protocol with minor modifications. In brief, the cells were washed twice with cold cell staining buffer (BioLegend, #420 201), resuspended in annexin V binding buffer (BioLegend, #422 201) to which FITC annexin V and 7‐AAD were both added at 1:20 dilution, and incubated for 15 min at RT in the dark. Then, the cells were washed twice with annexin V binding buffer and flow cytometry was performed in CytoFLEX (Beckman Coulter). Flowjo (BD Biosciences, V.10) was used to analyze the data. The live cells were negative for both annexin and 7‐AAD, the early apoptotic cells were positive for annexin V but negative for 7‐AAD, and the late apoptotic cells were positive for both annexin V and 7‐AAD.

### Transmission Electron Microscopy (TEM)

The airway organoids were first fixed with 2.5% glutaraldehyde in PBS overnight at 4 °C and washed 3 times with PBS. The organoids were further fixed with 1% osmium tetroxide for another 1 h at 4 °C and washed again as above. The organoids were then passed through a graded series of 50%, 70%, 80%, and 90% ethanol, followed by 2 washes of 100% ethanol, with 10 min per step. The organoids were further passed through 100% acetone twice for 10 min each to enhance dehydration. The dehydrated organoids were immersed in a series of graded epoxy resin and acetone, including 25% of epoxy resin in acetone for 30 min at RT, 50% of epoxy resin in acetone for 4 h at RT, and 100% of epoxy resin overnight at 4 °C. To promote polymerization of the epoxy resin, the organoid samples were incubated at 37 °C for 24 h and then at 60 °C for an additional 48 h. The sample blocks were sectioned at 100 nm using a Leica UC7 and the sections were placed on copper grids. Finally, the airway organoid sections were stained with uranyl acetate for 20 min and lead citrate for 12 min, respectively. The images were captured using the FEI Tecnai G2 Spirit transmission electron microscope.

### Measurement of Ciliary Beating Activity

The methodology was adapted from previous research to determine the ciliary beating activity with some modifications.^[^ ^31^ ^]^ Briefly, the ciliary beating of airway organoids was first recorded using a Nikon Ti2‐E with a 100X oil immersion objective. The exposure time was set to 50 ms with 16‐bit depth for all the recorded images. A series of beating cilia images were acquired from each organoid at 1 ms intervals for a duration of 10 s.  Timelapse videos of ciliary beating were generated using NIS‐Elements software.

To quantitatively analyze ciliary beating activity, a MATLAB pipeline was developed to process the image sequences of beating cilia. The Bio‐formats Toolkit was used to load the ND2 format of the original image sequences. The first image was processed by detecting its features with the Speeded‐Up Robust Features (SURF) method.^[^ ^32^ ^]^ This process included converting the images to grayscale, adjusting the contrast, applying median filtering to reduce noise, and detecting the SURF features. Similar preprocessing steps were applied to each subsequent image in the sequence. By matching the SURF features of each current image to those of the first image, we were able to determine how each subsequent image has moved or transformed relative to the first image. Each image follows the same processing procedure.

The first step in the SURF algorithm is to convert the original image into an integral image. An integral image at a location (x, y) stores the sum of all pixel intensities above and to the left of that location.

(1) $$I_{\Sigma }\left(x,y\right)=\sum_{i=0}^{x}\sum_{j=0}^{y}I\left(i,j\right)$$

Then, the determinant of the Hessian matrix was used to detect the points of interest. The Hessian matrix captures the second‐order partial derivatives of the image intensity, which is useful for identifying regions with significant changes in intensity. The hessian matrix of each location is defined as follows:

(2) $$H\left(x,y,\sigma \right)=\left[\begin{array}{cc} L_{xx}\left(x,y,\sigma \right) & L_{xy}\left(x,y,\sigma \right)\\ L_{xy}\left(x,y,\sigma \right) & L_{yy}\left(x,y,\sigma \right) \end{array}\right]$$

Here, L_xy_, L_xy_, and L_yy_ are the convolutions of the image with Gaussian second‐order derivatives in the respective directions based on the integral image.

To identify key points, the determinant of the Hessian matrix was calculated. This determinant provides a measure of local changes in intensity and helps to identify regions of interest:

(3) $$\mathrm{Det}\left(H\right)\approx D_{xx}D_{yy}-{\left(0.9\times D_{xy}\right)}^{2}$$

By ranking the determinant, the most representative point in each image could be obtained. By aligning the point of each image, the geometric transformation matrix could be estimated based on the aligned features, and this matrix could be used to correct or align the current image to match the orientation and position of the first image using RANSAC.^[^ ^33^ ^]^ After alignment, an interactive interface was designed to manually select the area of beating cilia and document the changes in pixel values throughout the sequence.

### Isothermal Titration Calorimetry (ITC)

The molecular interaction was measured using ITC (MICROCAL PEAQ‐ITC Automated), in accordance with the manufacturer's protocol. Briefly, cigarette condensate and nicotine was first added to a 96 Deepwell polypropylene plate (LABSELECT, DWR‐96‐UC‐1000A) at the final concentration of 0.5% (v/v) and 0.1 mM in ddH2O, respectively. Then, NAC solution was prepared at a concentration of 1 mM in ddH2O. NAC titration was performed in cigarette condensate and nicotine in a stepwise manner for a total of 19 injections, respectively. The cell temperature was set to 25°C. The reference power and initial delay were set to 10 µCal s^−1^ and 60 s respectively. A string speed of 750 rpm was used for the ITC measurement. The binding constant was determined by matching the titration curve to a one‐site binding mode, using MicroCal PEAQ‐ITC Analysis Software. To measure the binding affinity of nicotine or NAC with nAChRs, recombinant human nAChRα5 (MedChemExpress, #HY‐P70340) or nAChRα7 (ImmunoClone, #ICA305Hu01) was added to the 96 Deepwell polypropylene plate at a final concentration of 2 ug mL^−1^, and the titration was performed using nicotine or NAC at a concentration of 1 mM, as described above. To investigate the effect of NAC on the binding of nicotine to nAChRα5 or nAChRα7, NAC was added to nicotine in a stepwise manner until the 2 molecular interactions reached thermal equilibrium, as previously demonstrated. This NAC‐nicotine mixture was subsequently used for a secondary titration by continuously adding the mixture to nAChRα5 or nAChRα7 and the thermodynamic parameters was measured.

### UV–Vis Spectroscopy

All the reagents were diluted in ddH2O, including cigarette condensate (1:50 dilutions), NAC (10 mM), nicotine (1 mM), nAChRα5 (0.2 ug mL^−1^), and nAChRα7 (0.2 ug mL^−1^). Titration was performed by adding NAC to diluted cigarette condensate, NAC to nicotine, nAChRα5 to nicotine, and nAChRα7 to nicotine in a stepwise manner, respectively. UV–Vis absorption spectra were acquired for each step of the titration using a UV–Vis spectrophotometer (Thermo Fisher Evolution 201/220). To investigate the effect of NAC on the binding capacity of nicotine to nAChRs, NAC was first added to nicotine in a stepwise manner until the binding of these 2 molecules reached equilibrium as determined by the UV–Vis spectrophotometer.The mixture of NAC and nicotine was then added to nAChRs as described above and the change in absorbance was determined.

### Molecular Docking

Molecular docking was performed using the Glide module in Schrodinger 2018 software. The crystal structures of nAChRα5 (UniProt ID: P30532) and nAChRα7 (PDB ID: 8CAU) were obtained from the AlphaFold Protein Structure Database (https://alphafold.ebi.ac.uk/) and RCSB protein Data Bank (https://www.rcsb.org/), respectively. These structures were then assigned protonation states using Epik at a target pH range of 7.0 ± 2.0, subsequent to which structure optimization and energy minimization were carried out. These structures were assigned protonation states using Epik at a target pH value of 7.0 ± 2.0, followed by structure optimization and energy minimization. All ligands were pretreated with LigPrep using the OPLS‐2005 force field. The extra precision of the Glide module was employed for ligand docking and analysis of the results.

### Measurement of ROS

The levels of ROS in both airway organoids and airway organoid cells were determined using the ROS Assay Kit (Solarbio, #CA1410), in accordance with the manufacturer's protocol. Briefly, the organoids were first incubated in dichlorodihydrofluorescein diacetate (DCFH‐DA) solution at a final concentration of 10 µmol L^−1^ at 37 °C for 30 min, followed by 3 washes. The organoids were then transferred to Rosup (1:1000 dilutions in normal saline), nicotine (5 mM in normal saline), NAC (5 mM in normal saline), a mixture of nicotine and NAC (5 mM nicotine in combination with 5 mM NAC in normal saline), and normal saline, respectively. The airway organoids were incubated for another 30 min as above and the fluorescence was examined under the Nikon AX confocal microscope with 60X and 100X oil immersion objectives.

To determine the level of ROS in airway organoid cells, airway organoids were first dissociated into single cells using TrypLE (Gibco, #2 085 275) and then incubated with DCFH‐DA solution at a final concentration of 10 µmol L^−1^ under 37 °C for 30 min. After washing, the organoid cells were further incubated with Rosup, nicotine, NAC, a mixture of nicotine and NAC, and normal saline as described above, respectively. Finally, the ROS level in the airway organoid cells was determined using a fluorescence microplate (Tecan Infinite 200Pro).

### Statistical Analysis

Statistical analyses were performed using Prism (Version 9.4.1). All data were first checked for normality distribution  . Data were presented as mean and standard deviation (SD) in a line graph and histogram. Data were presented as median, interquartile range, minimum, and maximum in truncated violin plot. Each symbol represents a single individual. An alteration in ciliary beating frequency (%) for each group was normalized to its respective baseline ciliary beating frequency. Paired *t‐*test or Wilcoxon's signed‐rank test was used for the analysis of paired data, and unpaired *t‐*test or Mann‐Whitney's test was used for the analysis of unpaired data where appropriate. Ordinary analysis of variance (ANOVA) was used to determine the statistical difference across multiple groups. Statistically significant differences were indicated by the following asterisks: **** *p* < 0.0001; *** *p* < 0.001; ** *p* < 0.01; * *p* < 0.05. Two‐sided *p* < 0.05 was considered significant.

## Conflict of Interest

The authors declare no conflict of interest.

## Author Contributions

Y.C.Z. and S.H.M. conceived the study and designed experiments. Y.C.Z., J.X.Z., and S.Z. performed experiments. Y.C.Z, W.H., and Y.F.W. analyzed data. Z.C.Q. performed the molecular docking. Q.Y.T., Y.F.W, Y.D.C, Y.M.L, J.Q.H., and Y.Z. provided critical materials and consultation. S.H.M. supervised this work. Y.C.Z. prepared the draft manuscript. S.H.M. and D.D. edited the manuscript. All authors reviewed and approved the final manuscript.

## Supporting information



Supporting Information

Supplemental Video 1

Supplemental Video 2

Supplemental Video 3

Supplemental Video 4

Supplemental Video 5

Supplemental Video 6

Supplemental Video 7

Supplemental Video 8

Supplemental Video 9

Supplemental Video 10

Supplemental Video 11

## Data Availability

The data that support the findings of this study are available from the corresponding author upon reasonable request.
