# Supplementary material for: Identification of Nicotinic Acetylcholine Receptor for N‐Acetylcysteine to Rescue Nicotine‐induced Injury Using Beating Cilia in Primary Tissue Derived Airway Organoids
Source: Adv Sci (Weinh). 2024 Nov 24;12(1):2407054. doi: 10.1002/advs.202407054 (PMC11714201; doi:10.1002/advs.202407054)
Supplement: Supplementary file 1 — Supporting Information [file ADVS-12-2407054-s003.docx]

**Identification of Nicotinic Acetylcholine Receptor for N-acetylcysteine to Rescue Nicotine-induced Injury Using Beating Cilia in Primary Tissue Derived Airway Organoids**

Yichao Zheng^1,2^, Qinyong Tian^3^, Haowei Yang^1^, Yongde Cai^4^, Jiaxin Zhang^4^, Yifen Wu^5^, Shuo Zhu^6^, Zuocheng Qiu^7^, Yimin Lin^3^, Jiangquan Hong^3^, Yi Zhang^3^, David Dockrell^8^, and Shaohua Ma^1,2^

^1^Institute of Biopharmaceutical and Health Engineering, Tsinghua Shenzhen International Graduate School (SIGS), Tsinghua University, Shenzhen 518055, China. ^2^Precision Medicine and Healthcare Research Centre, Tsinghua-Berkeley Shenzhen Institute (TBSI), Tsinghua University, Shenzhen 518055, China. ^3^Department of Cardiothoracic Surgery, Zhangzhou Affiliated Hospital of Fujian Medical University, Zhangzhou 363000, China. ^4^Institute of Biopharmaceutical and Health Engineering, State Key Laboratory of Chemical Oncogenomics, Shenzhen International Graduate School, Tsinghua University, Shenzhen 518055, China. ^5^Department of Internal Medicine, Zhangzhou Affiliated Hospital of Fujian Medical University, Zhangzhou 363000, China. ^6^Key Laboratory of Rubber-Plastics, Ministry of Education/Shandong Provincial Key Laboratory of Rubber and Plastics, Qingdao University of Science and Technology, Qingdao 266042, China. ^7^Guangdong Provincial Key Laboratory of Speed Capability Research, Jinan University, Guangzhou, 510632, China. ^8^Department of Respiratory Medicine and MRC Centre for Inflammation Research, University of Edinburgh, Edinburgh, United Kingdom.

Corresponding author: Shaohua Ma ([ma.shaohua@sz.tsinghua.edu.cn](mailto:ma.shaohua@sz.tsinghua.edu.cn))

**Figure S1** Primary tissue-derived airway organoids from 8 different donors expressing acetylated-α-tubulin in cilia. Scale bar, 50μm.

**Figure S2** Primary tissue-derived airway organoids from different donors expressing MUC5AC, cytokeratin-5, acetylated-α-tubulin, and α-smooth muscle actin. Abbreviations: MUC5AC, mucin-5AC. Scale bar, 50μm.

**Figure S3** Primary tissue-derived airway organoids from different donors consistently exhibit apical-out polarization. Abbreviations: ZO-1, zonula occludens-1; DAPI, 4',6-diamidino-2-phenylindole. Scale bar, 50μm.

**Figure S4** TEM image of axoneme in cilia from different airway organoids. Abbreviations: TEM, transmission electron microscopy.


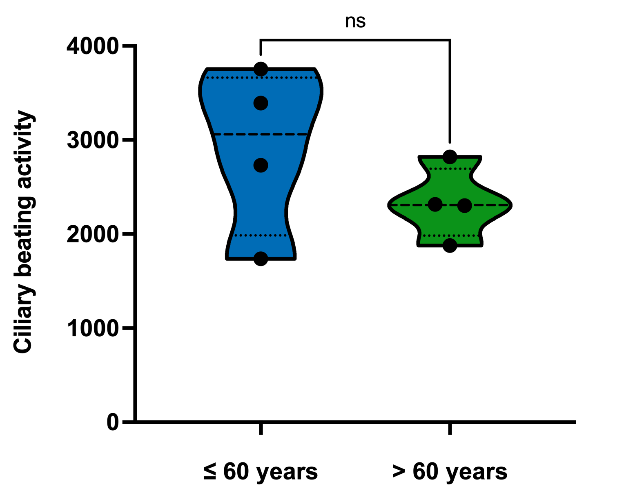

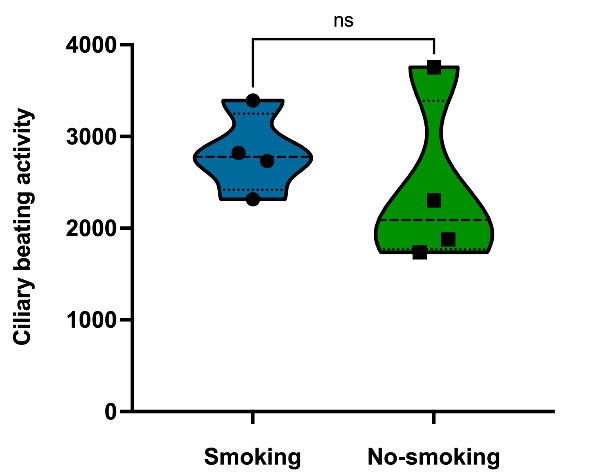


**B**

**A**

**Figure S5** Ciliary beating activity of different donors. A) A comparison of ciliary beating activity between donors aged ≤ 60 years and those aged > 60 years yielded a P value of 0.4857. B) A comparison of ciliary beating activity between donors with and without a history of smoking yielded a P value of 0.3429. The present analysis includes 8 donors. The data are presented as median and interquartile and Mann-Whitney test is used for statistical analysis. ns., not statistically significant difference.

**Figure S6** Cigarette condensate decreases ciliary beating frequency in a concentration-dependent manner over the 9-hour observation period. The data are presented as mean and SD for each group (n=3). Ordinary ANOVA was used to determine the statistical difference, with a P value of 0.5245, 0.0005, and p < 0.0001 at different time points (3h, 6h, 9h), respectively. Abbreviations: ANOVA, analysis of variance; SD, standard deviation.

**Figure S7** Impairment of ciliary beating activity is an early indicator of airway injury. Cigarette condensate induces a more pronounced reduction in ciliary beating activity compared to the control group (n=3). Data are presented as mean and SD. Unpaired *t* test test is used for statistical analysis, with a P value of <0.0001. The percentage of dead cells increased in the cigarette condensate-treated organoids 5 hours after the impairment of ciliary beating functionality. Scale bar, 50 μm. Abbreviations: PI, propidium iodide; SD, standard deviation.

**Figure S8** Cigarette condensate induces apoptosis in HBECs. A) Dynamic change in the proportions of live and apoptotic cells between different groups. B) Summary data of the proportions of live and apoptotic cells at 9 hours between different groups (n=3). The data are presented with mean and SD. The statistical difference between different time points is determined with paired t test. **** p<0.0001, *** p<0.001, ** p<0.01, * p<0.05. Abbreviations: HBECs, human bronchial epithelial cells; SD, standard deviation.

**Figure S9** Airway is resistant to cigarette condensate-induced apoptosis. Live (green) and dead (red) staining of airway organoids exposed to different concentrations of cigarette condensate for 9 hours in different groups. Scale bar, 50μm. Abbreviations: PI, propidium iodide.

**Figure S10** ALI culture of HBECs is susceptible to the gaseous phase of cigarette smoke. We exposed ALI culture of HBECs to smoke and burned the cigarette once every hour. We determined the proportion of live and apoptotic cells at different time points. The data are presented as mean and SD. Abbreviations: ALI, air-liquid interface; HBECs, human bronchial epithelial cells; SD, standard deviation.


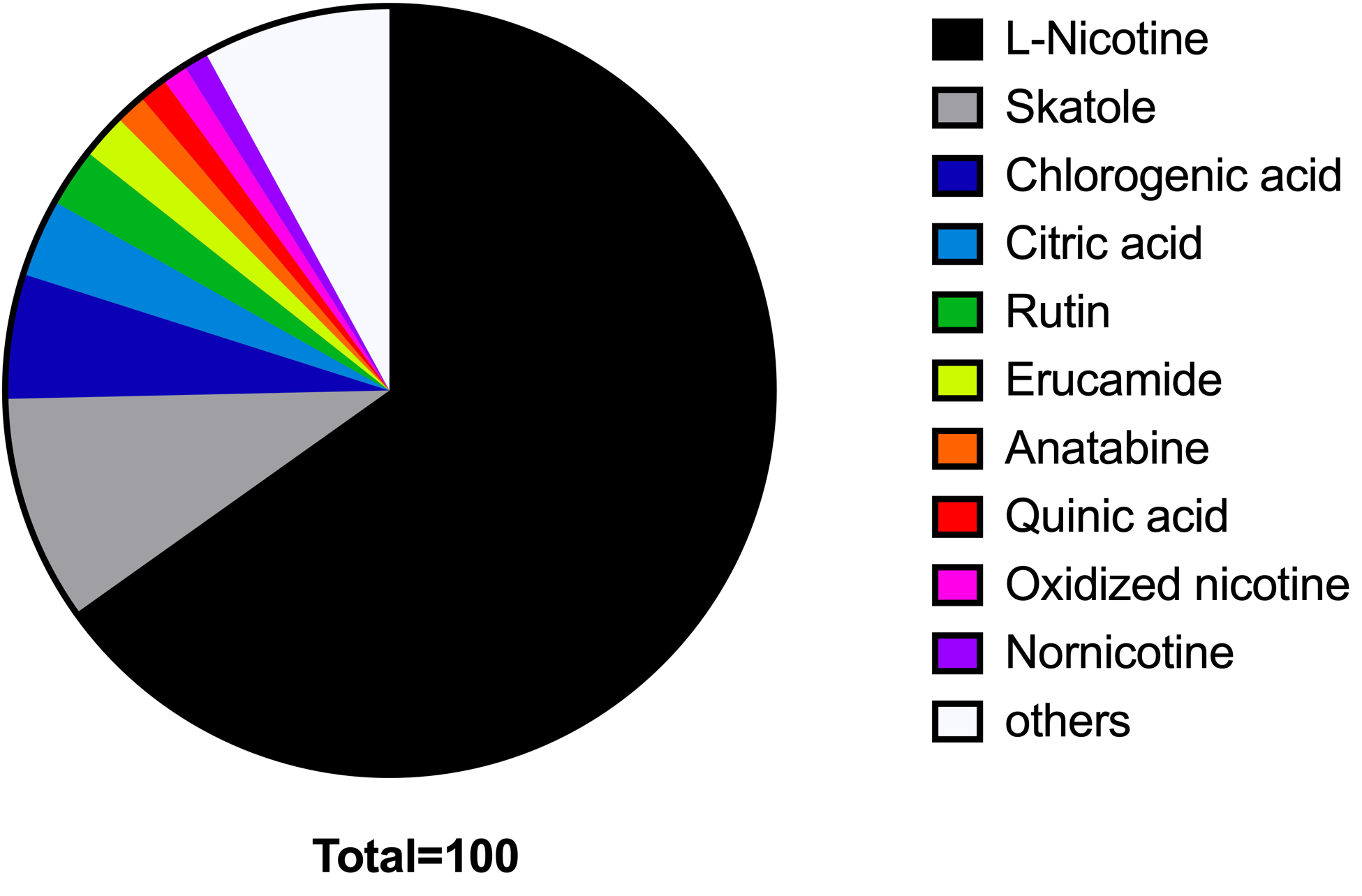


**Figure S11** Components of cigarette condensate determined by LC-MS. Abbreviations: LC-MS, liquid chromatography-mass spectrometry.

**Figure S12** NAC aerosol prevents cigarette smoke-induced ciliary dysfunction. A) The representative data of the ciliary beating activity between the two groups at different time points. The ciliary beating activity is reflected in the amplitude of the signal fluctuation. B) The summary data for the alteration in ciliary beating frequency between the two groups at the end of the experiment (3h). The truncated violin plot represents the median, interquartile range, minimum and maximum. An unpaired *t* test is used to determine the statistical significance, with a P value of < 0.0001. Each group contains 6 individual data (n=6). Abbreviations: GS, gaseous phase; NAC, N-acetylcysteine.

**Figure S13** NAC liquid prevents cigarette smoke-induced ciliary dysfunction in a concentration-dependent manner. A) The representative data of the ciliary beating activity among different groups at different time points. B) The summary data for the alteration in ciliary beating frequency between different groups at the end of the experiment (3h) with a P value of < 0.0001; the data are presented with the median, interquartile range, minimum and maximum. Each symbol represents an individual. Each group contains 6 single individuals (n=6). The statistical significance between different groups was determined by ordinary ANOVA. Abbreviations: ANOVA, analysis of variance; GS, gaseous phase; NAC, N-acetylcysteine.

**Figure S14.** The effect of NAC on cigarette condensate-induced ciliary dysfunction. A) NAC liquid prevents the CS-induced weakening of ciliary beating signals in a concentration-dependent manner over the 9-hour observation period. B) The frequency of ciliary beating increases with higher concentrations of NAC at the end of the experiment (9h). The data are presented as mean and SD for each group (n=6). Each symbol represents an individual. The statistical significance between different groups is determined using ordinary ANOVA, with a P value of < 0.0001. Abbreviations: CS, cigarette condensate; NAC, N-acetylcysteine; SD, standard deviation; ANOVA, analysis of variance.

**Figure S15** NAC prevents cigarette condensate-induced apoptosis. We exposed HBEC cultures to cigarette condensate with or without NAC treatment (n=3). We harvested cells from each group after 9 hours of treatment and determined the proportions of live and apoptotic cells. The data are presented with median and interquartile. The statistical difference across multiple groups is determined with ordinary ANOVA with a P value of 0.0051. Abbreviations: HBEC, human bronchial epithelial cell; NAC, N-acetylcysteine; ANOVA, analysis of variance.

**Figure S16.** NAC has no apparent toxicity on the ciliary beating activity of the airway organoids. A) NAC of different concentrations do not significantly decrease ciliary beating activity at different time points over the 9-hour period. B) The quantitative analysis of ciliary beating activity reveals no discernible pattern in the concentration- or time-dependent weakening of ciliary beating activity at the end of the experiment (9h). Data are presented with mean and SD for each group (n=6). Each symbol represents a single individual. Ordinary ANOVA is used to determine the statistical difference between different groups, with a P value of < 0.0001. Abbreviations: NAC, N-acetylcysteine; SD, standard deviation; ANOVA, analysis of variance.

**Figure S17** Primary tissue-derived airway organoids from different donors co-expressing acetylated-α-tubulin and nAChRα5 in cilia (left figure). Zo-1 is presented underneath the cilia, suggesting an “apical-out” polarity (right figure). Abbreviations: ZO-1, zonula occludens-1; DAPI, 4',6-diamidino-2-phenylindole.

**Figure S18** nAChRα7 is abundantly distributed in the apical membrane of airway organoids. Scale bar, 50μm. Abbreviations: DAPI, 4',6-diamidino-2-phenylindole.

**Figure S19** Super-resolution images of ciliary structure, and the expression of nAChRα5 (A) and nAChRα7 (B). Scale bar, 20 μm.

**Figure S20** Molecular interaction of nicotine with nicotinic receptors. nAChRα5 (top figure) and nAChRα7 (bottom figure) solutions at a concentration of 0.2 ug/ml were added to nicotine solution (1 mM) in a dose-escalation manner. The binding of nicotine to nicotinic receptors is reflected by the change in absorption spectra between the wavelengths of 230 nM and 270 nM.

**Figure S21** Interaction between NAC and nAChRs. NAC solution at a concentration of 1 mM was added to nAChRα5 (2 ug/ml) and nAChRα7 (2 ug/ml) in a dose-escalation manner, respectively. The alterations in DP and ΔH in response to the molecular interactions were determined. NAC has a binding affinity of 61.5e^-6^ and 9.57e^-6^ for nAChRα5 and nAChRα7, respectively. Abbreviations: NAC, N-acetylcysteine; DP, differential power; ΔH, enthalpy of reaction.

**Figure S22** Molecular interactions between NAC and nicotine. NAC solution (10 mM) was added to nicotine solution (1 mM) in a dose-escalation manner. The binding of these two molecules can change the absorption spectra, especially between the wavelengths of 230 nM and 270 nM. Abbreviations: NAC, N-acetylcysteine.

.

**Figure S23** NAC interferes with the interactions between nicotine and nicotinic receptors. NAC (10 mM) was added to nicotine (1 mM) in a dose-escalation manner as described above, until the interactions of these two molecules reach reached equilibrium. This mixture of nicotine and NAC was then added to nAChRα5 and nAChRα7 respectively, and the normalized absorbance was determined. In the control group, the same volume of ddH2O was added to nAChRα5 and nAChRα7 respectively, and the normalized absorbance was measured. Abbreviations: NAC, N-acetylcysteine.

**Figure S24** NAC shows binding capacity to cigarette condensate as evidenced by the change in the thermodynamic parameters (left figure) and absorption intensity (right figure) during the molecular interactions. The titration procedure is described in Experimental Section. Abbreviations: NAC, N-acetylcysteine; DP, differential power; ΔH, enthalpy of reaction.

| **No.** | **Age** | **Gender** | **Smoking** | **Diagnosis** |
| --- | --- | --- | --- | --- |
| 1 | 60 | Male | 30 years | Left lower lobe lung adenocarcinoma (p-T1aN0M0, stage IA1) |
| 2 | 65 | Female | No | Right upper lobe lung adenocarcinoma (p-T1cN0M0, stage IA3) |
| 3 | 70 | Male | 30 years | Right middle lobe lung adenocarcinoma (p-T1cN0M0, stage IA3) |
| 4 | 45 | Female | No | Left upper lobe lung adenocarcinoma (p-T1miN0M0, stage IA1) |
| 5 | 70 | Female | No | Left upper lobe lung adenocarcinoma (p-T1cN0M0, stage IA3) |
| 6 | 61 | Female | No | Right lower lobe bronchiectasia |
| 7 | 54 | Male | 30 years | Right upper lobe lung adenocarcinoma (p-T1bN0M0, stage IA2) |
| 8 | 67 | Male | 40 years | Left lower lobe lung adenocarcinoma (p-T1bN0M0, stage IA2) |
| 9 | 53 | Female | No | Left upper lobe lung adenocarcinoma (p-T1bN0M0, stage IA2) |
| 10 | 72 | Male | 40 years | Left lower lobe lung squamous cell carcinoma (c-T2bN3M0, stage IIIB) |

Table S1. Main clinical characteristics of the participants

Table S2. Analysis of compounds in cigarette condensate

| **Compound** | **Formula** | **Molecular weight** | **Peak area** | **Peak area (%)** |
| --- | --- | --- | --- | --- |
| L-Nicotine | C10H14N2 | 162.11542 | 1.75E+11 | 65.17 |
| Skatole | C9H9N | 131.07341 | 2.55E+10 | 9.48 |
| CHLOROGENIC ACID | C16H18O9 | 354.09434 | 1.41E+10 | 5.25 |
| Citric acid | C6H8O7 | 192.02603 | 8.79E+09 | 3.27 |
| Rutin | C27H30O16 | 610.15211 | 6.62E+09 | 2.46 |
| Erucamide | C22H43NO | 337.3337 | 5.12E+09 | 1.90 |
| Anatabine | C10H12N2 | 160.10005 | 3.45E+09 | 1.28 |
| Quinic acid | C7H12O6 | 192.06227 | 3.18E+09 | 1.18 |
| Oxidized nicotine | C10H14N2O | 178.11029 | 2.79E+09 | 1.04 |
| Nornicotine | C9H12N2 | 148.0999 | 2.78E+09 | 1.03 |
| L-Phenylalanine | C9H11NO2 | 165.07874 | 2.17E+09 | 0.81 |
| Caffeic acid | C9H8O4 | 180.04126 | 1.96E+09 | 0.73 |
| Scopoletin | C10H8O4 | 192.04192 | 1.73E+09 | 0.64 |
| Corchorifatty acid F | C18H32O5 | 328.22441 | 1.65E+09 | 0.61 |
| kaempferol-3-O-rutinoside | C27H30O15 | 594.15742 | 1.43E+09 | 0.53 |
| 2-Pyrrolidinecarboxylic acid | C5H9NO2 | 115.06348 | 1.14E+09 | 0.43 |
| Gluconic acid | C6H12O7 | 196.05725 | 9.90E+08 | 0.37 |
| 2-Isopropylmalic acid | C7H12O5 | 176.06717 | 9.67E+08 | 0.36 |
| Choline | C5H13NO | 103.10002 | 9.53E+08 | 0.35 |
| Abietic Acid | C20H30O2 | 302.22386 | 8.87E+08 | 0.33 |
| Curcumenol | C15H22O2 | 234.16152 | 8.67E+08 | 0.32 |
| N-acetyltryptophan | C13H14N2O3 | 246.0999 | 7.74E+08 | 0.29 |
| trans-3-Indoleacrylic acid | C11H9NO2 | 187.06319 | 5.75E+08 | 0.21 |
| Curcumenol | C15H22O2 | 234.16144 | 5.28E+08 | 0.20 |
| Arachidonic acid | C20H32O2 | 304.2402 | 4.62E+08 | 0.17 |
| N-acetylphenylalanine | C11H13NO3 | 207.08847 | 4.47E+08 | 0.17 |
| Azelaic acid | C9H16O4 | 188.10386 | 3.77E+08 | 0.14 |
| Isoquercitrin | C21H20O12 | 464.09413 | 3.55E+08 | 0.13 |
| Linolenic acid ethyl ester | C20H34O2 | 306.25511 | 2.83E+08 | 0.11 |
| Xylose | C5H10O5 | 150.0515 | 2.78E+08 | 0.10 |
| L-Tryptophan | C11H12N2O2 | 204.08975 | 2.57E+08 | 0.10 |
| Kaempferol | C15H10O6 | 286.04686 | 2.32E+08 | 0.09 |
| DL-4-Hydroxyphenyllactic acid | C9H10O4 | 182.05693 | 2.15E+08 | 0.08 |
| 3-phenyllactic acid | C9H10O3 | 166.06177 | 1.50E+08 | 0.06 |
| 3-O-Feruloylquinic acid | C17H20O9 | 368.11043 | 1.49E+08 | 0.06 |
| Propamocarb | C9H20N2O2 | 188.15233 | 1.36E+08 | 0.05 |
| Hyperoside | C21H20O12 | 464.09477 | 1.36E+08 | 0.05 |
| Gentisic acid | C7H6O4 | 154.02534 | 8.97E+07 | 0.03 |
| α-Cyperone | C15H22O | 218.16657 | 8.73E+07 | 0.03 |
| Astragalin | C21H20O11 | 448.09892 | 8.69E+07 | 0.03 |
| 3,4-di-O-caffeoylquinic acid | C25H24O12 | 516.1263 | 8.30E+07 | 0.03 |
| (-)-Caryophyllene oxide | C15H24O | 220.18221 | 8.03E+07 | 0.03 |
| Isoalantolactone | C15H20O2 | 232.14592 | 7.91E+07 | 0.03 |
| abscisic acid | C15H20O4 | 264.13579 | 7.19E+07 | 0.03 |
| Sucrose | C12H22O11 | 342.11571 | 6.94E+07 | 0.03 |
| N-Acetyl-D-alloisoleucine | C8H15NO3 | 173.10414 | 6.80E+07 | 0.03 |
| (±)9-HpODE | C18H32O4 | 312.22966 | 6.71E+07 | 0.02 |
| feruloyltyramine | C18H19NO4 | 313.13103 | 6.23E+07 | 0.02 |
| Atractylenolide II | C15H20O2 | 232.14589 | 4.96E+07 | 0.02 |
| Lariciresinol 4-O-glucoside | C26H34O11 | 522.20949 | 4.70E+07 | 0.02 |

Table S3. Interactive activity between different molecules

| **No.** | **Molecule 1** | **Molecule 2** | **Affinity constant, KD (M)** |
| --- | --- | --- | --- |
| 1 | Nicotine | nAChRα5 | 4.99e^-6^ |
| 2 | Nicotine | nAChRα7 | 42.7e^-6^ |
| 3 | N-acetylcysteine | Nicotine | 278e^-6^ |
| 4 | N-acetylcysteine | Cigarette condensate | 25.4e^-6^ |
| 5 | Mixture of nicotine and N-acetylcysteine | nAChRα5 | N.A. |
| 6 | Mixture of nicotine and N-acetylcysteine | nAChRα7 | N.A. |

**Supplemental Video 1 —** Beating cilia produce different motions of airway organoids.

**Supplemental Video 2 —** Airway organoids show apical-out polarity. Cilia are stained with acetylated-α-tubulin.

**Supplemental Video 3 —** Gaseous phase of cigarette smoke impairs ciliary beating activity. Scale bar, 25 μm.

**Supplemental Video 4 —** Cigarette condensate impairs ciliary beating activity in a concentration- and time-dependent manner. Scale bar, 25 μm.

**Supplemental Video 5 —** Impairment of ciliary beating functionality occurs earlier than the appearance of apoptosis.

**Supplemental Video 6 —** Inhaled NAC aerosol prevents smoking-induced ciliary dysfunction in ALI culture of airway organoids. Scale bar, 25 μm. Abbreviations: NAC, N-acetylcysteine; ALI, air-liquid interface.

**Supplemental Video 7 —** The liquid form of NAC prevents cigarette smoke-induced ciliary dysfunction in a concentration-dependent manner in ALI culture of airway organoids. Scale bar, 50 μm. Abbreviations: NAC, N-acetylcysteine; ALI, air-liquid interface.

**Supplemental Video 8 —** NAC treatment prevents cigarette condensate-induced ciliary dysfunction in a concentration-dependent manner. Scale bar, 50 μm. Abbreviations: NAC, N-acetylcysteine.

**Supplemental Video 9 —** NAC has no apparent side effect on the ciliary beating functionality of airway organoids. Scale bar, 50 μm. Abbreviations: NAC, N-acetylcysteine.

**Supplemental Video 10 —** NAC prevents purified nicotine-induced ciliary dysfunction. Scale bar, 25 μm. Abbreviations: NAC, N-acetylcysteine.

**Supplemental Video 11 —** Adiphenine as an nAChR inhibitor can suppress ciliary beating activity in a concentration-dependent manner. Scale bar, 25 μm.
